# Nontoxic Dual-Function Probe for Ratiometric Oxygen Sensing and Cellular Imaging Based on Ir^III^–Eu^III^-Functionalized SiO_2_ Particles

**DOI:** 10.1021/acsomega.6c00449

**Published:** 2026-05-21

**Authors:** Felipe S. M. Canisares, Alessandra M. G. Mutti, João Antonio O. Santos, Alessandro B. S. Garcia, Marian R. Davolos, Ana M. Pires, Sergio A. M. Lima

**Affiliations:** † 28108 São Paulo State University (UNESP), Institute of Chemistry, Av. Prof. Francisco Degni, 55 - Jardim Quitandinha, Araraquara 14800-900, São Paulo, Brazil; ‡ University of São Paulo (USP), Institute of Chemistry, Av. Prof. Lineu Prestes, 748 - Butantã, São Paulo 05508-900, Brazil; § School of Technology and Sciences, São Paulo State University (UNESP), R. Roberto Símonsen, 305 - Centro Educacional, Presidente Prudente 19060-900, São Paulo, Brazil; ∥ São Paulo State University (Unesp), Institute of Biosciences, Humanities and Exact Sciences, R. Cristóvão Colombo, 2265 - Jardim Nazareth, São José do Rio Preto 15054-000, São Paulo, Brazil

## Abstract

Hypoxia, characterized by low tissue oxygen concentration, is a common feature of diseases such as cardiac ischemia, inflammation, brain disorders, and solid tumors. Consequently, oxygen sensing has gained attention in diagnostics. In this study, we synthesized luminescent silica (SiO_2_) particles functionalized with a bimetallic Ir^III^–Eu^III^ complex for ratiometric oxygen sensing in biological media. Spherical SiO_2_ particles (288 nm) were prepared via the sol–gel method to support and transport the water-insoluble luminescent complex. Then, the Ir^III^–Eu^III^ complex was grafted stepwise, yielding the final hybrid SiO_2_–Eu^III^Ir^III^ that exhibits a broad excitation band from 250 to 550 nm, yellow emission (by combining the green emission of Ir^III^ complex and red emission from Eu^III^ ion), and a negative surface potential in water, allowing stable suspension. Oxygen sensing experiments revealed a nonlinear sensing response with 70.5% sensitivity to oxygen concentration. Toxicity assays using Huh-7.5 cells showed no cytotoxicity in concentrations between 1.56 and 400 μg mL^–1^. Confocal microscopy confirmed particles internalization, keeping their luminescent properties in the green and red regions, both emissions were detected separately after exciting at 488 nm. These results demonstrate the potential of SiO_2_–Eu^III^Ir^III^ particles as a ratiometric luminescent probe for oxygen detection and cell labeling in biological applications.

## Graphical abstract

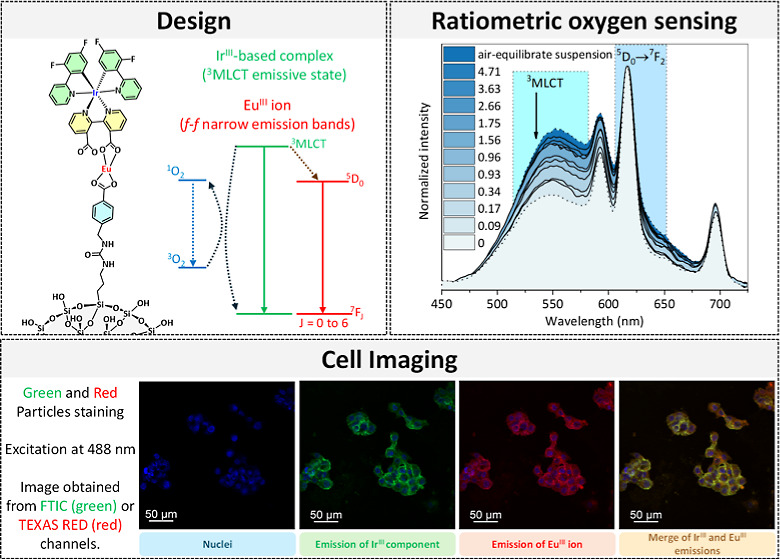

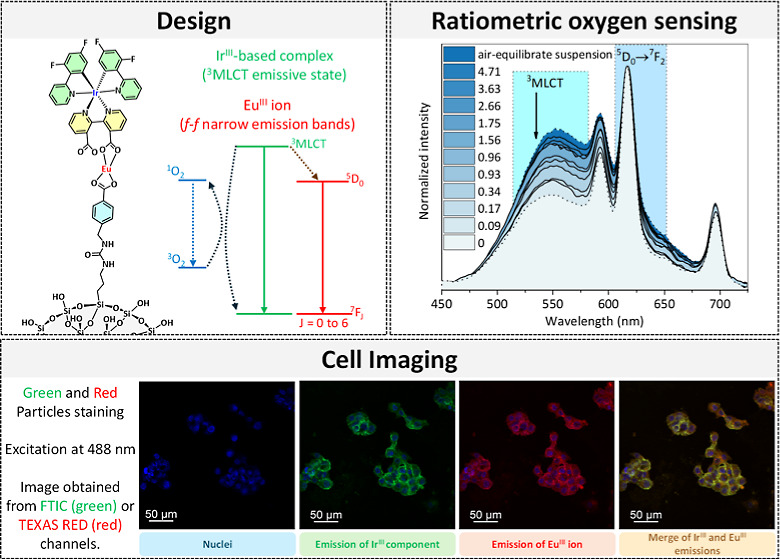

## Introduction

Molecular oxygen (O_2_) is of great importance in aerobic organisms because it acts as an electron acceptor molecule in the energy production process (ATP).^1^ Hence, our organism seeks to maintain constant levels of this molecule in tissues to meet energy needs. Values different from the ideal may indicate or even stimulate pathological processes such as inflammatory diseases, cardiac ischemia, respiratory abnormalities, and cancer.^2,3^ Despite the great advances in the understanding and treatment of cancer, it remains a major challenge for global health once this disease represents the second cause of death in the world.^4,5^ One of the main reasons for the high death rates is the low specificity and sensitivity of methods for the early diagnosis of such disease.^6–8^ Usually, the quantification of O_2_ in the biological medium is performed through sampling or chemical analysis.^9^ Thus, continuous monitoring requires the development of minimally or noninvasive techniques for both *in situ* and *in vivo* analysis.^10–12^ Among the techniques currently explored for such quantification, simple and low-cost electrochemical sensors stand out.^9^ Other more sophisticated techniques have also been used for this purpose, such as electronic paramagnetic resonance,^13^ functional magnetic resonance,^14^ positron emission tomography (PET),^15^ pulsed oximetry,^16^ and luminescent imaging.^17^ In this last technique, sensors based on phosphorescent materials have made an important contribution.

Oxygen detection by phosphorescence suppression is a direct analysis, measured through the deactivation of the triplet-emitting state of luminophores, after a photochemical process characterized by the collision of the luminescent probe with molecular oxygen species.^10,18^ The luminophore absorbs photons that pass to an excited state of singlet character (S_n_) with higher energy. After internal conversion (IC), these photons arrive at the lowest energy excited level with the same spin multiplicity (S_1_), and through intersystem crossing (ISC) mechanism, they populate an emitter level of triplet character (T_1_). This general process is represented in Figure 1.

**Figure 1:**
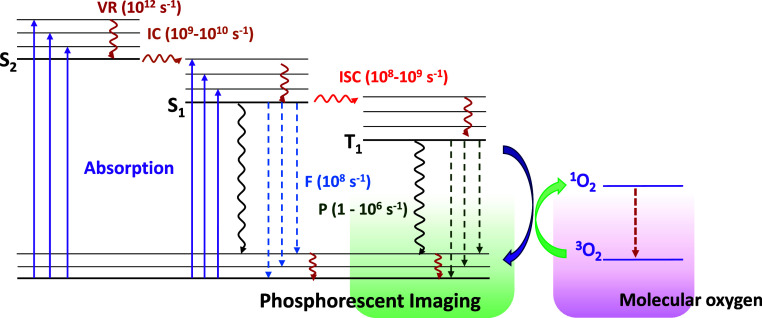
Schematic representation of the deactivation of triplet states of phosphorescent molecules by oxygen using the simplified Jablonski diagram. VR: Vibrational Relaxation; IC: Internal Conversion; ISC: Intersystem Crossing; F: Fluorescence; P: Phosphorescence. The typical time scale of each event is indicated in the Figure 1.^19^ Full arrow: absorption; wavy arrow: nonradiative process; dashed arrow: emission. Adapted with permission from Imran, M.; Chen, M. S. Self-sensitized and reversible O_2_ reactivity with bisphenalenyls for simple, tunable, and multicycle colorimetric oxygen-sensing films. ACS Applied Materials & Interfaces 2021, 14 (1), 1817–1825. Copyright © 2021 American Chemical Society.^20^

Molecular oxygen exhibits a triplet paramagnetic ground state (^3^O_2_), the same multiplicity as the excited state of the probe, which accepts the energy of the excited molecule generating a highly reactive and short-lived singlet-character excited state (^1^O_2_). This singlet oxygen species returns to the ground state quickly after interacting with solvent molecules, oxidating neighboring chemical structures, or emitting a photon resulting in a weak phosphorescence emission at 1270 nm,^21^ which has been studied in trials for photodynamic therapy (PDT).^22^

When rationalizing the choice of ideal probes for *in vivo* intracellular studies, several factors must be considered, such as:^10^ (i) high absorption and emission efficiencies; (ii) emission ranging from visible to near-infrared (NIR), allowing imaging with color variation and easy tissue penetration in *in vivo* assays; (iii) moderately long lifetime (>1 μs), allowing lifetime imaging; (iv) high photostability; (v) high efficiency in the cell internalization process; vi) specific subcellular localization; and vii) low cytotoxicity and phototoxicity.

Among the phosphorescent materials used for oxygen detection, complexes based on the Ir^III^ ion have demonstrated great potential for application and meet most of the above requirements mainly because of their flexible molecular design.^23,24^ Although highly indicated for biomedical applications in the measurement of oxygen concentration, most of the Ir^III^ complexes studied for this purpose emit from a single emitting level,^25^ resulting in only one emission band. Single-band luminescent sensors have limitations of application, the main one being their high dependence on the concentration of the probe used. Therefore, if part of the applied probe is metabolized and/or lost in the process, the entire method is invalidated, as the intensity of the emission is strictly dependent on the concentration.^26^ Therefore, dual emission probes have been the subject of research in the process of measuring and quantifying dissolved oxygen in biological media, since these two components present distinct behavior under oxygen concentration variation.^27^ The ratio of dual or multiple emission bands in the sensing process makes the probe self-calibrating, resulting in greater analysis sensitivity, both graphically, through the emission rate, and by image, through visual analysis.^28–30^ Despite the well-known advantages of ratiometric luminescence sensing, such as improved accuracy and reduced influence of external factors, its application for oxygen detection remains relatively limited. This is mainly associated with the challenge of designing systems that exhibit two well-resolved and independently responsive emission signals while maintaining efficient interaction with oxygen molecules. In addition, the development of stable materials with appropriate photophysical properties and controlled energy-transfer processes remains a significant challenge, which has limited the broader implementation of ratiometric oxygen sensors in practical applications.^31^

Knowing that complexes based on Ir^III^ ion are highly qualified for molecular oxygen sensing, a strategy that we used here was the coordination of Ir^III^-based complexes into Europium­(III) ion to create a ratiometric probe composed of Ir^III^–Eu^III^ bimetallic complexes. Typically, lanthanide ion pairs, such as Eu^III^–Tb^III^, are employed in ratiometric approaches due to their well-defined and distinguishable emission bands.^32^ Only recently have bimetallic Ir^III^–Eu^III^ complexes been explored as potential systems for ratiometric temperature sensing.^33^ Such a system will present emission of two distinct bands that respond differently to the oxygen concentration and thus allow ratiometric detection. In 2016, A. J. Metherell and co-workers^34^ showed that an Ir^III^–Eu^III^ bimetallic complex has selectively quenched the Eu^III^ emission against VO (2-diisopropylaminoethyl ethyl methylphosphonate) molecule; they also performed ratiometric sensing of this analyte with changes in the overall luminescence color. Another study developed by H. Jhang and co-workers reported^35^ the use of a polymeric structure composed by Ir^III^ and Eu^III^ for temperature sensing in living cells and zebrafish using ratiometric and phosphorescence lifetime imaging microscopy. Other classes of materials have also attracted considerable interest for oxygen sensing applications, particularly metal–organic frameworks (MOFs), due to their highly tunable porous architectures, which enable efficient diffusion and interaction with oxygen molecules.^36,37^

Oxygen sensing in biological media using complexes based on Ir^III^ and Ln^III^ is challenging because most are nonsoluble in aqueous medium. To overcome this problem, biocompatible particles have been used as molecular carriers to maintain the hydro stabilization of the suspension and to help the internalization of such materials through the cell membranes.^38^ One of the most used carriers is silica particles. Silica is an inorganic polymer that acts perfectly as a host matrix for molecules and biomolecules, creating promising materials devoted to innumerable applications in a variety of science fields. Silica particles are transparent in a wide spectral region, and the presence of ionized groups on the surface adds a hydrophilic property to them, and consequently, they become more stable in aqueous medium.^39,40^ Furthermore, the synthesis of silica can be achieved by the sol–gel method that allows grafting a huge number of luminescent units in one single particle, and it results in intensification of emission of the hybrid material, disregarding cross relaxation and concentration quenching.^41^

The present report introduces the development of a luminescent probe composed of silica particles decorated with Ir^III^–Eu^III^ heterobimetallic complexes produced by a step-by-step approach for dual application: ratiometric oxygen sensing and cell imaging. For this, silica particles were synthesized by Stöber method and then functionalized with carboxylic groups. After this, Eu^III^ ions were coordinated following by reaction with [Ir­(dfppy)_2_(bpdc)]. The structural and spectroscopic properties of this luminescent probe were evaluated and studied in exploratory tests as a biological luminescent stain in living cells and as oxygen sensing in solution.

## Experimental Section

### Materials and Reagents

Ammonium hydroxide (Synth, 24–26%); ethanol (Synth, 99.5%); tetraethyl orthosilicate (TEOS) (Aldrich, 99%); acetonitrile (Synth, 99.5%); dichloromethane (Synth, 99.5%); 3-(triethoxysilyl)­propyl isocyanate (IPTES) (Aldrich, 95%); and 4-(aminomethyl)­benzoic acid (abac) (Aldrich, 97%). All reagents and solvents were used without further purification.

### Instrumentation

FTIR spectra were recorded using an FTIR IRAffinity-1 Shimadzu spectrometer within the 4000–400 cm^–1^ range, recorded using 128 scans, resolution of 1 cm^–1^, in ATR mode. Transmission electron microscopy (TEM) images were collected from a JEM2100 LaB6 (TEM) equipped with chemical analyses (energy dispersive spectroscopy—EDS). Confocal microscopy images were performed on a Carl Zeiss model LSM 800 with Airyscan, equipped with three detection channels, four laser lines (405, 488, 561, and 640 nm), and brightfield image scanning. Elemental analyses (CHN) were collected in a PerkinElmer 2400 series ii. Luminescent measurements were performed using a Horiba Jobin Yvon Fluorolog-3 Spectrofluorometer, model Fluorolog-3 −221, equipped with a continuous 450 W-xenon lamp, double excitation and emission monochromator, and an R 928 Hamamatsu photomultiplier. All spectra were corrected using a detector mode correction. The absolute emission quantum yield values were recorded using an integrating sphere with an absolute error of 10% in its value. Thermal analyses were performed using TA Instruments model SDTQ600 equipment.

### Step I—Silica (SiO_2_) Particles Synthesis

In a beaker 11.2 mL of distilled water, 2.7 mL of concentrated ammonium hydroxide, and 94.8 mL of ethanol were added and stirred at 40 °C for 5 min. Thereafter, 7.2 mL of TEOS (31.9 mmol, Aldrich, 99%) was added dropwise and stirred for 90 min. Then, the mixture rested overnight at room temperature. The material was centrifuged and washed with ethanol, acetonitrile, and dichloromethane, respectively, and oven-dried at 70 °C for 24 h, yielding a mass of 4.4374 g. FTIR (KBr cm^–1^): 3215 ν_as_(O–H); 1069 ν_as_(Si–O–Si); 952 δ­(Si–OH); 794 ν_s_(Si–O–Si); 469 δ­(Si–O–Si).

### Step II—Functionalization with NCO Groups (SiO_2_–NCO)

A mass of 3.0319 g of SiO_2_ particles was first oven-dried at 100 °C for 1 h and suspended in 150 mL of ethanol in an ultrasound bath for 15 min. A solution of 11.96 mL (49.3 mmol) of IPTES (3-(triethoxysilyl)­propyl isocyanate) in 80 mL of ethanol was dropwise added to the SiO_2_ suspension, and the reaction run under magnetic stirring for 12 h at room temperature. The material was centrifuged and washed with ethanol, acetonitrile, and dichloromethane, respectively, and oven-dried at 70 °C for 24 h, yielding a mass of 2.7995 g. FTIR (KBr cm^–1^): 3314 ν_as_(O–H); 1056 ν_as_(Si–O–Si); 945 δ­(Si–OH); 794 ν_s_(Si–O–Si); 445 δ­(Si–O–Si).

### Step III—Functionalization with COOH Groups (SiO_2_–COOH)

A mass of 2.0147 g of SiO_2_–NCO particles was oven-dried at 100 °C for 1 h and suspended in 50 mL of DMSO in an ultrasound bath for 15 min. Then, 5.1 mL (6.9 mmol) of tetraethylammonium (TEA) 20% was added followed by 1.0298g (6.6 mmol) of 4-(aminomethyl)­benzoic acid. The solution was stirred at room temperature for 20 h. The material was centrifuged and washed with ethanol, acetonitrile, and dichloromethane, respectively, and oven-dried at 70 °C for 24 h, yielding a mass of 1.7572 g. FTIR (KBr cm^–1^): 3334 ν_as_(O–H); 1059 ν_as_(Si–O–Si); 945 δ­(Si–OH); 794 ν_s_(Si–O–Si); 445 δ­(Si–O–Si). Elemental analysis: C: 3.13%, H: 1.55%, N: 0.72%.

### Step IV—Coordination of the Eu^III^ Ion (SiO_2_–Eu^III^)

A mass of 0.5249 g of SiO_2_–COOH was oven-dried at 100 °C for 1 h and suspended in 50 mL of ethanol using an ultrasound bath. The solution was stirred at room temperature, and 0.0483 g (1.2 mmol) of NaOH previously dissolved in water was added to deprotonate the carboxylic acid groups. Then, 34 mL (1.6 mmol) of EuCl_3_ 0.049 mol·L^–1^ was added dropwise and stirred at room temperature for 24 h. The material was centrifuged and washed with ethanol, acetonitrile, and dichloromethane, respectively, and oven-dried at 70 °C for 24 h, yielding a mass of 0.5787 g. FTIR (KBr cm^–1^): 3354 ν_as_(O–H); 1066 ν_as_(Si–O–Si); 945 δ­(Si–OH); 794 ν_s_(Si–O–Si); 445 δ­(Si–O–Si). Elemental analysis: C: 1.75%, H: 1.45%, N: 0.25%.

### Step V–Ir^III^–Eu^III^ Bimetallic Complex Formation (SiO_2_–Eu^III^Ir^III^)

A mass of 0.3070 g of SiO_2_–Eu^III^ was oven-dried at 100 °C for 1 h and suspended in 50 mL of methanol. Then, a methanolic solution prepared with 0.0217 g (0.03 mmol) of [Ir­(fdppy)_2_(bpdc)] was added into the reaction flask. [Ir­(dfppy)_2_(bpdc)] was synthesized and characterized by us in a previous work,^42^ where dfppy is the cyclometallating ligand 2-(2,4-difluorphenyl)­pyridine, and bpdc is the bridging ligand 2,2′-bipyridine-3,3′-dicarboxylic acid. The solution was kept under stirring at room temperature for 24 h. The material was centrifuged and washed with ethanol, acetonitrile, and dichloromethane, respectively, and oven-dried at 70 °C for 24 h, yielding a mass of 0.2890 g. FTIR (KBr cm^–1^): 3354 ν_as_(O–H); 1079 ν_as_(Si–O–Si); 945 δ­(Si–OH); 794 ν_s_(Si–O–Si); 445 δ­(Si–O–Si). Elemental analysis: C: 2.84%, H: 1.52%, N: 0.55%.

### Oxygen Sensing Measurements

A suspension of SiO_2_–Eu^III^Ir^III^ particles was prepared in water at a concentration of 0.0944 mg·mL^–1^ using ultrasound bath. Then, the solution was kept under stirring and nitrogen gas was purged to vary the oxygen concentration in the solution. The concentration of dissolved oxygen (DO) in ppm was monitored using a DO sensor with screw-type connector from Hannah instruments. Emission spectra were recorded with oxygen concentration variation in a PerkinElmer LS55 fluorimeter equipped with a Xe lamp (9.9 W) and an R928 PMT photomultiplier. The data were analyzed by spectral changes, graphical plots (ratio between Ir^III^ and Eu^III^ emission and dissolved oxygen concentration, DO in ppm), and the two-site Stern–Volmer equation.

### Cytotoxicity Assays in Huh 7.5 Cells

Cytotoxicity assays were performed using the MTT method^43^ in Huh-7.5 cells (human hepatocellular carcinoma cell line). Cells were cultured in Dulbecco’s modified Eagle’s medium (DMEM, Sigma-Aldrich) supplemented with 10% fetal bovine serum, 1% penicillin–streptomycin, and nonessential amino acids. Subsequently, cells were trypsinized, counted using a Neubauer chamber, and seeded into 96-well plates at a density of 2.5 × 10^5^ cells per well. The plates were incubated for 24 h at 37 °C in a humidified atmosphere containing 5% CO_2_ to allow cell adhesion. After incubation, the cells were exposed to hybrid suspensions prepared in DMEM at concentrations ranging from 400 to 1.56 μg mL^–1^ for 24 h. Negative control (NC) consisted of cells incubated with DMEM alone. Following the exposure period, the medium was replaced with a freshly prepared MTT solution (3-(4,5-dimethylthiazol-2-yl)-2,5-diphenyltetrazolium bromide, 1 mg mL^–1^) and incubated for 4 h. The resulting formazan crystals were dissolved in isopropyl alcohol. Absorbance was measured at 570 nm using a microplate reader (UT-200A). Cell viability was calculated according to eq 1, where Abs_sample_ represents the mean absorbance of treated samples at each concentration and Abs_NC_ corresponds to the mean absorbance of the negative control. All tests were performed in triplicate.

1 $$Cellviability\left(\%\right)=\frac{{Abs}_{sample}}{{Abs}_{NC}}x100$$

### Sample Preparation for Confocal Microscopy Analysis

The coverslips were assembled for confocal microscopy by incubating cells with the particles in solution at 50 μg mL^–1^ in DMEM culture medium for 2 h. Such concentration was chosen based on previous toxicity analysis. After the medium was removed, the cell nuclei were stained with Hoechst nuclear dye for 15 min. Then, cells were washed three times with PBS and fixed with 2.5% glutaraldehyde in PBS.

## Results and Discussion

### Structural Characterization

The samples were synthesized as shown in the synthetic procedure detailed in Scheme 1, the functionalization steps were based on ref [^44^].

**Scheme 1:**
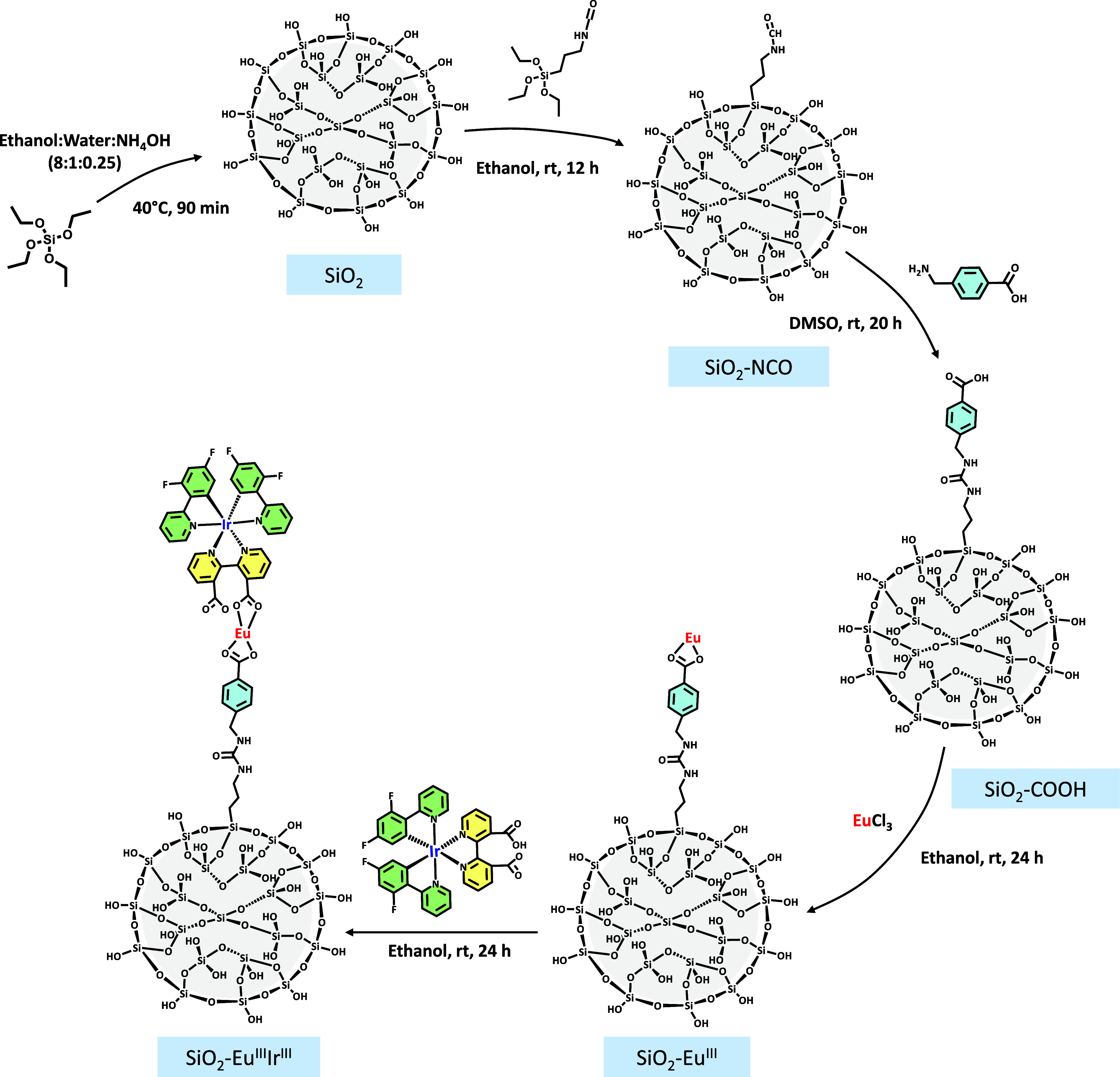
Schematic Representation of SiO_2_–Eu^III^Ir^III^ Step-by-step Synthesis. The Molecules and the Particles are out of Scale and Merely Represent an Illustration of the Formation of the Hybrid

One of the most important characteristics of the sol–gel methodology is the size and shape homogeneity of the synthesized particles. To analyze this aspect, TEM was performed on the final hybrid (SiO_2_–Eu^III^Ir^III^), Figure 2A. From the image obtained, it is possible to observe that spherical particles were formed with an average diameter of 288.8 ± 24.9 nm, as measured using the ImageJ software.^45^ Figure 2A shows a representative TEM image of the SiO_2_–Eu^III^Ir^III^ particles, and Figure 2B shows the histogram of particle size distribution estimated by counting 100 particles.

**Figure 2:**
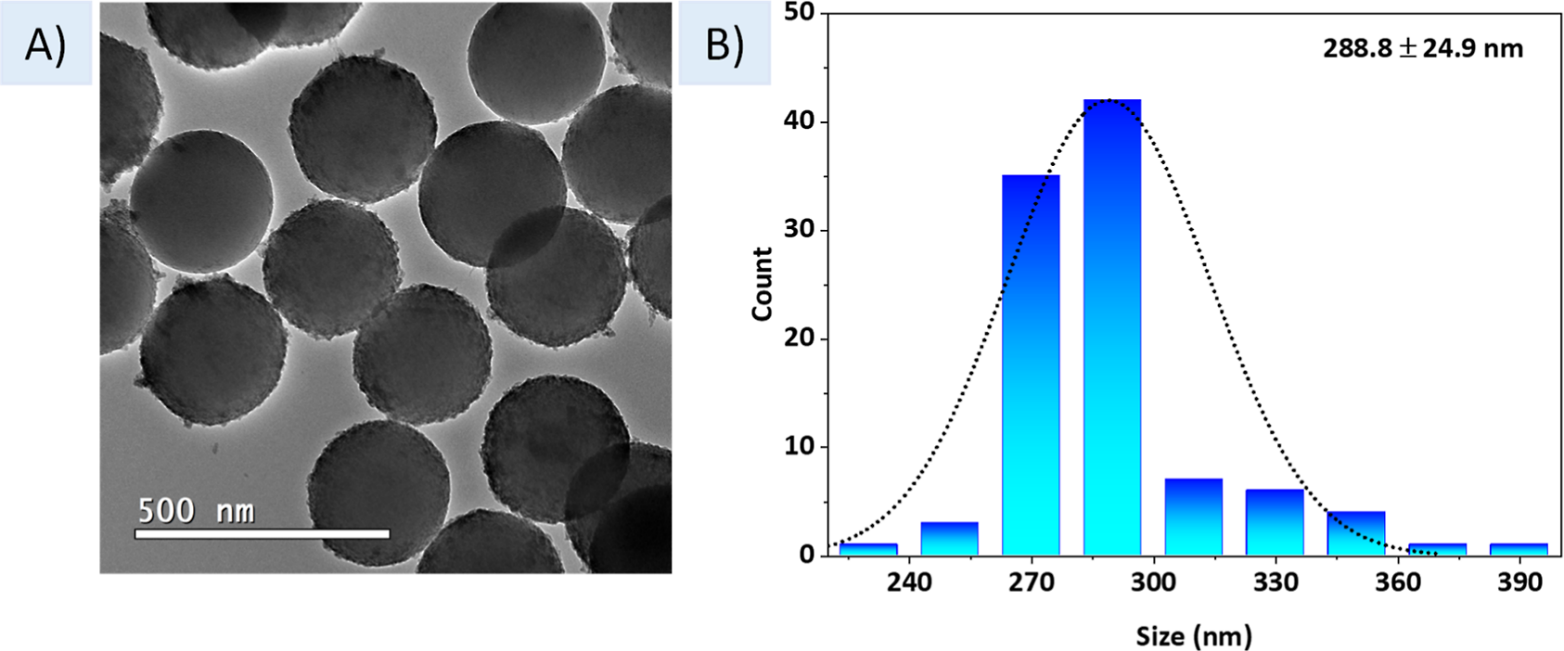
(A) TEM image of SiO_2_–Eu^III^Ir^III^ particles and (B) histogram of the size distribution.

To evaluate the thermal stability and the surface modification of the samples related to each synthesis step, thermogravimetric analysis (TGA) was performed in the range of 30 to 1000 °C, Figure S1. Two main thermal events occurring in different temperature ranges were observed through the differential thermogravimetric curves (DTG). While the first event (up to ∼200 °C) is associated with the loss of physiosorbed water or solvent molecules, the second (between ∼200 to 800 °C) is linked to silica dehydroxylation and combustion of the anchored organic matter. In general terms, a gradual increase in mass loss occurs during the second thermal event as seen in Table S1, indicating that the surface modification steps were carried out successfully as loading organic molecules on the surface of silica resulted in greater mass loss by thermodecomposition.

By FTIR spectroscopy, Figure S2, as the functionalization steps progress, the band centered at 1061 cm^–1^ associated with the antisymmetric stretching (ν_as_) mode of the Si–O–Si group shifts to higher energy, and it is placed at 1079 cm^–1^ in the final hybrid, SiO_2_–Eu^III^Ir^III^. On the other hand, the band assigned to the symmetric stretching (ν_s_) mode of the Si–O–H group shifted to lower energy, from 952 cm^–1^ in the SiO_2_ particles to 945 cm^–1^ in the final hybrid. Other bands associated with the inorganic silica matrix did not undergo displacement, such as ν_s_(Si–O–Si) at 799 cm^–1^ and δ­(Si–O–Si) at 467 cm^–1^. In the first two functionalization steps (SiO_2_–NCO and SiO_2_–COOH), no distinct bands related to the grafted organic moieties were detected, likely due to the low amount of such groups on the surface of the particles compared to the core of silica, thus the intense vibrational modes of the silica framework mask the contributions from any organic vibration. After coordination of Eu^III^ to the carboxylic groups (SiO_2_–Eu^III^), a band at 1398 cm^–1^ appears, assigned to the symmetric stretching mode of the carboxylate (COO^–^) group, indicating deprotonation of the COOH groups and coordination to the Eu^III^ center. Following incorporation of the Ir^III^ complex (SiO_2_–Eu^III^Ir^III^), a band at 1603 cm^–1^ is observed, which is attributed to the antisymmetric stretching of the carboxylate group and the C=N stretching vibration of the Ir^III^ complex used to sum to the coordination sphere of Eu^III^.

The degree of functionalization of the particles was determined by elemental analysis. Using this technique, it was possible to determine the mass percentage of carbon and nitrogen, and by their ratio, to validate the proposed structure, as well as to determine the amount of organic matter grafted onto the silica particles. First, it is important to emphasize that the synthetic methodology (step-by-step) produced the proposed structures because the experimental C/N ratio is close to the theoretical one for the last three hybrids (SiO_2_–COOH, SiO_2_–Eu^III^, and SiO_2_–Eu^III^Ir^III^), as depicted in Table 1 and Figure S3. The SiO_2_–Eu^III^ intermediate sample exhibited an experimentally determined C/N ratio of 8.16, which is higher than expected. This discrepancy is likely attributed to the coordination of ethanol molecules with the Eu^III^ center, which contributes additional carbon to the sample, as detailed in Table 1, where a C/N ratio of 8 is observed after the coordination of two ethanol molecules. Furthermore, water molecules are also expected to coordinate to the Eu^III^ ion; however, their presence does not affect the C/N ratio. The final hybrid, SiO_2_–Eu^III^Ir^III^, was theoretically predicted to possess a C/N ratio of 8, based on the assumption of two iridium complexes per europium­(III) ion. Nevertheless, the experimentally measured C/N ratio was found to be 6.02, indicating a lower carbon content than initially designed. We attribute this to the steric hindrance encountered by the Ir^III^- complexes as they coordinate to the Eu^III^ ion priorly grafted onto the surface. The steric effect renders the surrounding –COOEu^III^ groups less accessible for coordination with additional Ir^III^ complexes, thereby resulting in an anchoring rate lower than anticipated.^64^ Additionally, the Ir^III^ complex used in this study contains two distinct carboxyl groups, which can be easily coordinated to two neighbors Eu^III^ ions, thereby contributing to the reduction of the C/N ratio, so the experimental ratio of Ir^III^ and Eu^III^ is between 1 and 2. The degree of functionalization was determined using SiO_2_–COOH sample, and through the percentage of carbon or nitrogen, it was calculated the number of carboxyl groups grafted on the surface of the particles. The percentage of carbon in the SiO_2_–COOH sample is 3.13 wt %, which is equivalent to 260.8 mmol of carbon considering 100 g of sample. Because the number of C atoms for each organic chain bearing 1 carboxylate group is 12, the concentration of carboxylic groups on the silica surface is 21.7 mmol in 100 g, which corresponds to 0.21 mmol g^–1^ (carboxylic groups by silica mass). It was also calculated by the percentage of nitrogen (0.72 wt %), and the estimated value was found to be 0.25 mmol g^–1^. These values are in the same order of magnitude as those found during functionalization processes previously published by our research group.^36,46,47^ Detailed calculations regarding the C/N ratio and the degree of functionalization are provided in Section III of the Supporting Information.

**Table 1:** Percentage Values and C/N Ratio between Carbon and Nitrogen Calculated (Theo.) and Found (Exp.) for SiO_2_–COOH, SiO_2_–Eu^III^, and SiO_2_–Eu^III^Ir^III^

	C wt %	N wt %	*R*_C/N__(exp.)_	*R*_C/N__(theo.)_
SiO_2_–COOH	3.13	0.72	5.09	6
SiO_2_-Eu^III^	1.75	0.25	8.18	6
SiO_2_-Eu^III^(C_2_H_5_OH)_2_				8
SiO_2_-Eu^III^Ir^III^ (2 Ir^III^ Complex)	2.84	0.55	6.03	8
SiO_2_-Eu^III^Ir^III^ (1 Ir^III^ Complex)				7.7

Seeking an ideal material for biological applications, surface potential is an essential parameter that drives the formation of a colloidal suspension^36^ and in some cases the fate of the particles inside the cell.^42^ The stability of the suspension depends on the high repulsion between particles of the same charge; thus, the higher the value of the zeta potential, negative or positive, the more stable the colloidal suspension will be. On the other hand, if the particles exhibit a neutral potential value or close to zero, they begin to form clusters or agglomerates and then precipitate. The surface potential was monitored for all samples in PBS buffer (pH 7.64) at 0.01 mol L^–1^, Figure S4. The SiO_2_ particles showed a negative potential due to the presence of ionized OH groups on the surface. The p*K*_a_ values of these groups are approximately 4;^48^ thus, at a pH of 7.64, the silanol group (Si–OH) is deprotonated, and thus creates a negative potential, predominantly composed of Si–O^–^ species. After the first and second functionalization steps, i.e., SiO_2_–NCO and SiO_2_–COOH, the surface potential slightly decreases because of the reaction of some ionized silanol groups, which decreases the negative charge on the surface. The load variation is small in the first two stages, indicating a low degree of functionalization as expected from the previous TGA and FTIR results, besides the carboxylic group added also present a negative charge, so it is not expected a significant change in the charge of the functionalized silica. After coordinating the Eu^III^ ion, the surface potential displays a larger change, from −18.93 mV in SiO_2_–COOH to −10.74 mV in SiO_2_–Eu^III^, due to the 3+ charge of the Eu^III^ ion. After the last synthetic step, the coordination of the Ir^III^ complexes, the negative potential increases again up to −14.2 mV, as the carboxyl groups of the Ir^III^ complex neutralized the positive charge of Eu^III^.

To evaluate the hydrodynamic size of SiO_2_–Eu^III^Ir^III^ particles in aqueous and DMEM suspensions, Dynamic Light Scattering (DLS) measurements were performed. The average hydrodynamic diameters were 1332 ± 65 nm in water and 2324 ± 506 nm in DMEM. These values are larger than those obtained by TEM because DLS measures the solvated particles and possible aggregates in suspension, whereas TEM provides the size of the dry inorganic core.^49,50^ For the SiO_2_–Eu^III^Ir^III^ aqueous suspension, time-dependent DLS measurements were performed at 30 min intervals over 1 h. The hydrodynamic size remained nearly constant (Figure S5A), whereas the polydispersity index (PDI) increased (Figure S5B), suggesting possible sedimentation and/or aggregate formation. To better understand the sedimentation behavior of the particles in suspension, the colloidal stability of the silica suspension was evaluated (0.1 mg mL^–1^) in aqueous medium and DMEM was evaluated by monitoring the absorbance at 400 nm over time using UV–vis spectroscopy.^51,52^ At this wavelength, the signal arises predominantly from light scattering by the dispersed silica particles, since no significant electronic absorption occurs. The stability index (SI) was calculated as the ratio between the absorbance at time *A*_t_ and the initial absorbance *A*_0_ (SI = *A*_t_/*A*_0_). Measurements were performed at 5 min intervals over a period of 2 h. Under these conditions, SI values close to unity indicate a stable dispersion, whereas decreasing values reflect nanoparticle aggregation and sedimentation.^53,54^ After 2 h, both suspensions exhibited relatively high SI values (0.77 in aqueous medium and 0.90 in DMEM, Figure S5C), indicating good colloidal stability. The slightly higher stability observed in DMEM is likely associated with the presence of electrolytes in the medium, which may contribute to stabilizing colloidal dispersion.

## Photoluminescence Study

The photoluminescence spectra of the SiO_2_–Eu^III^ and SiO_2_–Eu^III^Ir^III^ hybrids were measured in the solid state at room temperature and are shown in Figure 3. The SiO_2_, SiO_2_–NCO, and SiO_2_–COOH samples were also monitored, and the emission and excitation spectra are found in Figures S6 and S7, respectively.

**Figure 3:**
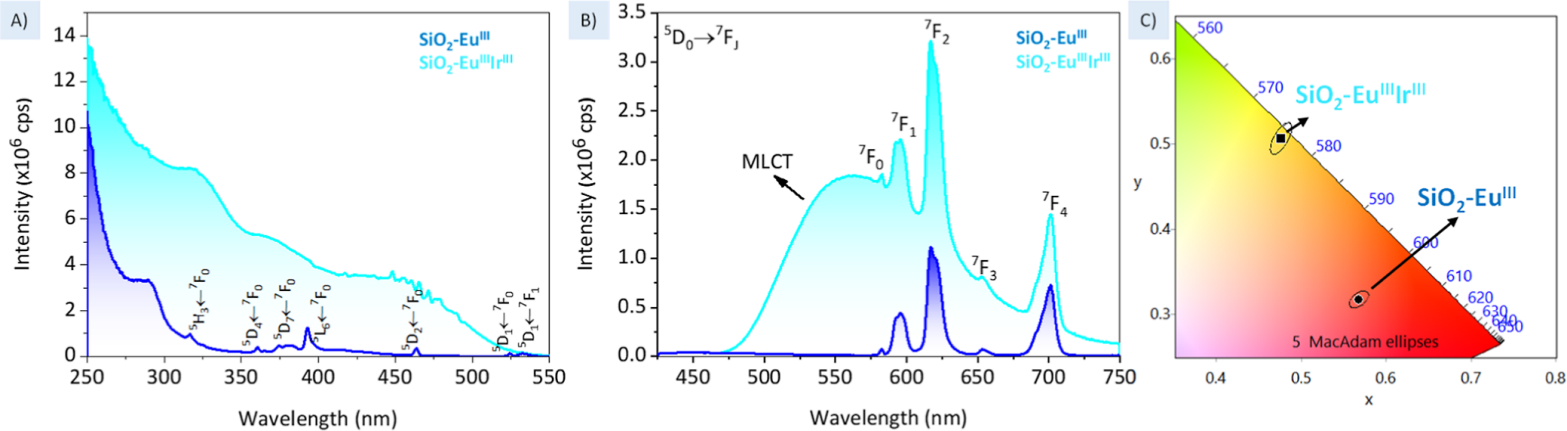
(A) Excitation and (B) emission spectra of SiO_2_–Eu^III^ (dark blue) and SiO_2_–Eu^III^Ir^III^ (light blue), and (C) color diagram of SiO_2_–Eu^III^ and SiO_2_–Eu^III^Ir^III^ samples measured in the solid state at room temperature. All measurements were carried out with a bandpass of 2.5 nm for both excitation and emission, with an increment of 0.5 nm and an integration time of 0.5 s λ_ex_ 393 nm and λ_em_ 617 nm.

The excitation spectrum of SiO_2_–Eu^III^ exhibits a wide band with high intensity extending from 250 to 325 nm, which is attributed to the S_1_ ← S_0_ absorption of the organic functionalized carboxylate chain. In addition, narrow *4f-4f* excitation bands of the Eu^III^ ion are observed, such as ^5^H_3_ ← ^7^F_0,_^5^D_4_ ← ^7^F_0_, ^5^L_7_ ← ^7^F_0_, ^5^L_6_ ← ^7^F_0_, ^5^D_2_ ← ^7^F_0_, ^5^D_1_ ← ^7^F_0_, and ^5^D_1_ ← ^7^F_1_ at 318, 362, 382, 393, 464, 524, and 532 nm, respectively.^55^ For the SiO_2_–Eu^III^-Ir^III^ sample, the visualization of Eu^III^ *4f-4f* transitions is difficult because the absorption band of the Ir^III^ complex used as ligand extends from 250 nm to approximately 550 nm and is associated with allowed transitions, thus covering up the weak Eu^III^ transitions. This excitation profile in the final hybrid is preferred for bio application, as excitation at lower energy can minimize damage to biological tissues. Table 2 presents the photophysical parameters derived from the emission spectra and lifetime decay of SiO_2_–Eu^III^ and SiO_2_–Eu^III^Ir^III^, which were obtained upon excitation at 393 nm and emission at 617 nm for Eu^III^ component, and upon excitation at 369 nm and emission at 558 nm for the iridium component. The emission decay curves of the Eu^III^ and Ir^III^ components for SiO_2_–Eu^III^ and SiO_2_–Eu^III^Ir^III^ are presented in the Supporting Information (Figures S8–S19).

**Table 2:** Photophysical Data From SiO_2_–Eu^III^ and SiO_2_–Eu^III^Ir^III^ Samples Measured in Powder, as Well as in Aqueous, Deuterated Aqueous, and DMEM Suspension^a^ ^b^ ^c^

	lifetime Eu^III^ component [μs]	lifetime Ir^III^ component [ns]	*A*_rad_ [s^–1^]	*A*_nrad_ [s^–1^]	Φ_Eu_^Eu^ [%]	Φ [%]	λ_dom_ [nm]	color purity [%]
SiO_2_-Eu^III^ (powder)	106.3	-	322	9084	3.4	-^a^	619	95
SiO_2_-Eu^III^Ir^III^ (powder)	139.8	-	327	6825	4.6	1.7 ± 0.2	576	67
SiO_2_-Eu^III^ (H_2_O)	100.4	-	361	9599	3.6	1.0 ± 0.1	470	17
SiO_2_-Eu^III^Ir^III^ (H_2_O)	192.8	21.2	196	5017	3.8	1.3 ± 0.1	577	82
SiO_2_-Eu^III^ (D_2_O)	215.4	-	137	4505	3.0	3.2 ± 0.3	599	43
SiO_2_-Eu^III^Ir^III^ (D_2_O)	588.3	45.0	199	1420	12.8	3.7 ± 0.4	587	89
SiO_2_-Eu^III^Ir^III^ (DMEM)	114.9	39.1	233	11144	2.0	-^a^	-^a^	-^a^
SiO_2_-Eu^III^Ir^III^ (H_2_O) degassed	217.5	15.3	-	-	-	-	578	85

a The Φ was too low to be accurately determined.

b The emission from DMEM overlaps with the nanoparticle emission.

c A_rad_ is the radiative decay rate of ^5^D_0_ of Eu^III^ ion, A_nrad_ is the non-radiative decay rate of Eu^III^ ion, Φ_Eu_^Eu^ is the intrinsic emission quantum yield, Φ is the absolute emission quantum yield, and λ_dom_ is the dominant emission wavelength.

The set of equations used to calculate these parameters is outlined in Section 2 of the Supporting Information. The lifetime decays for SiO_2_–Eu^III^Ir^III^ were fitted biexponentially, and to determine the average lifetime the following equation was applied^56^

2 $$<\tau >=\frac{\Sigma A_{i}\tau_{i}^{2}}{\Sigma A_{i}\tau_{i}}$$

in this equation, *A*_*i*_ is the pre-exponential factor or amplitude associated with each lifetime value (τ_*i*_). For the SiO_2_–Eu^III^ sample, a monoexponential fitting model was applied to both powder and aqueous solution measurements. In this system, the coordination environment of the Eu^III^ ion is more readily controlled, as only a carboxylate ligand is expected to coordinate to the metal center, with solvent molecules completing the coordination sphere. In deuterated aqueous suspension, water molecules may be displaced, leading to a heterogeneous coordination environment and, consequently, requiring bi- or multiexponential fitting models. For the SiO_2_–Eu^III^Ir^III^ sample, however, controlling the number of Ir^III^-based ligand complexes coordinating to each Eu^III^ ion is more challenging. This results in a heterogeneous system with multiple symmetry sites, characterized by different numbers of Ir^III^ complexes coordinated to the Eu^III^ ion, which in turn leads to distinct emission lifetimes, and thus requiring bi- or multiexponential fitting models.^57^ The emission profile of the SiO_2_–Eu^III^ sample in powder is dominated by narrow bands of the Eu^III^ ion, attributed to ^5^D_0_ → ^7^F_0_ (582 nm), ^5^D_0_ → ^7^F_1_ (595 nm), ^5^D_0_ → ^7^F_2_ (616 nm), ^5^D_0_ → ^7^F_3_ (653 nm) and ^5^D_0_ → ^7^F_4_ (701 nm). The intrinsic emission quantum yield (Φ_Eu_^Eu^) was determined to be 3.4%; this low value is attributed to water (and/or ethanol) molecules that complete the Eu^III^ coordination sphere until its saturation and promote vibronic deactivation of the excited states. Moreover, the dominant emission wavelength is centered at 619 nm with 95% color purity. The quenching of the ^5^D_0_ emissive state of Eu^III^ ion is also evidenced by the low lifetime value, 106.3 μs. After coordinating the Ir^III^ complex, in addition to the narrow *4f-4f* emission bands, a broad unstructured intense band dominates the emission spectrum that extends from 475 to 750 nm, characteristic of a big contribution of the ^3^MLCT state on the hybrid emissive state from the Ir^III^ moiety. As is typical for Ir^III^-based complexes, the photoluminescence arises from a mixture of triplet excited states. Accordingly, the overall triplet wave function (Ψ_T_) can be described as a linear combination of these states, as expressed by Ψ_T_ = aΨ­(^3^LC)+ bΨ­(^3^MLCT), where a and b are the corresponding normalization coefficients. Broad and unstructured emission bands indicates that the triplet metal-to-ligand charge-transfer (^3^MLCT) state has a much bigger weight in the mixture, whereas structured bands with well-defined vibronic progression originate from a bigger contribution of the ligand-centered (^3^LC) emissive states.^58^ As a result, a yellow perception is observed with a dominant emission wavelength of 576 nm and color purity of 67%. Furthermore, the intrinsic emission quantum yield increased compared to SiO_2_–Eu^III^, now being 4.6%, as shown in Table 2. Looking at the radiative (*A*_rad_) and nonradiative (*A*_nrad_) decay rate constants it is possible to see that the coordination of the Ir^III^ complex on Eu^III^ displaced some of the quenching water molecules, since the nonradiative decay rate constant decreased from 9084 s^–1^ to 6825 s^–1^. To further elucidate this behavior, the photophysical properties were investigated in H_2_O and D_2_O suspensions. The excitation and emission spectra are shown in Figures S20 and S21, and the corresponding photophysical parameters are summarized in Table 2. The number of coordinated water molecules (q) bound to Eu^III^ ions was estimated using the Horrocks equation^59^ and the ^5^D_0_ emission lifetimes (ms), $q=1.05\left[\tau_{H_{2}O}^{-1}-\tau_{D_{2}O}^{-1}\right]$ , for the systems before (SiO_2_–Eu^III^) and after coordination of the Ir^III^-based complex (SiO_2_–Eu^III^Ir^III^). The results reveal a decrease in the number of coordinated water molecules from 5.6 to 3.7 upon coordination. The noninteger values indicate a heterogeneous coordination environment, in which multiple Eu^III^ sites coexist, consistent with the multiexponential ^5^D_0_ decay profiles. Yet, the Δ*q* ∼ 2 suggest that two water molecules were substituted by an iridium complex that coordinates via the carboxylic group in a bidentate mode. This result agrees with the C/N ratio that indicate suggested a 1:1 ratio of Eu^III^/Ir^III^. Furthermore, the absolute emission quantum yield (Φ) of the SiO_2_–Eu^III^Ir^III^ was measured, yielding a value of 1.7% in powder and 1.3% in aqueous suspension upon excitation at 393 nm, which is close to values found on literature considering oxygen sensing probes based on lanthanide­(III) ions.^60^ The related bimetallic complex [{Ir­(dfppy)_2_(μ-bpdc)}_3_Eu_2_]­Cl_3_·nH_2_O·mCH_3_OH (dfppy = 2-(2,4-difluorophenyl)­pyridine; bpdc = 2,2′-bipyridine-3,3′-dicarboxylic acid) was previously reported, with overall emission quantum yields of 9.7% in dichloromethane, 6.6% in acetonitrile, and 4.4% in ethanol. The relatively lower Φ value observed here for the bimetallic structure grafted onto silica particles can be attributed to differences in the coordination environment of Eu^III^, particularly the smaller number of Ir^III^ complexes coordinated to the Eu^III^ center. This results in a coordination sphere containing a larger number of solvent or surface-related species capable of promoting nonradiative deactivation of the Eu^III^ excited state. In addition, the measurements reported here were performed in aqueous or solid media, where water molecules and molecular oxygen can further contribute to quenching of the emissive state.

The excitation and emission profiles were investigated in DMEM suspension (Figure S22), revealing spectral overlaps between the DMEM emission and that of SiO_2_–Eu^III^Ir^III^. Time-resolved measurements were therefore performed, and the Eu^III^ emission was selectively monitored. The emission lifetimes of both Eu^III^ and Ir^III^ components (Figures S16 and S17) were analyzed; compared to the aqueous suspension, an increase in the Ir^III^ emission lifetime and a decrease in the Eu^III^ emission lifetime were observed. The intrinsic Eu^III^ emission quantum yield (Φ_Eu_^Eu^) was determined to be 2.0%. The Φ was not evaluated due to the overlap between the fluorescence of the DMEM medium and the emission of the functionalized silica particles.

### Oxygen Sensing

Triplet emissive states are well-known for their high sensitivity to oxygen molecules. In this way, it is expected that the Ir^III^ moiety emission, in the SiO_2_–Eu^III^Ir^III^ system, undergoes an intensification as the oxygen content decreases, thus the Eu^III^ emission becomes less evident. However, since the system introduced here is composed of two distinct emission channels, and since they are connected by energy transfer, this mechanism must be considered to understand the spectral modification when oxygen-sensing measurements are performed. Therefore, the emission behavior of the *d-f* bimetallic hybrids measuring oxygen content is not obvious. The influence of oxygen molecules in the energy transfer process is evidenced by measuring the emission spectra of the hybrid varying the oxygen concentration. The emission spectra for oxygen-sensing measurements were recorded with a PerkinElmer LS55 fluorimeter (9.9 W Xe lamp, R928 PMT), adapted for collection during nitrogen purging. The excitation wavelength of 250 nm was chosen, corresponding to the maximum excitation intensity, as can be seen in Figure S20, ensuring efficient excitation and high-resolution emission data. The excitation spectrum of the aqueous suspension extends from 250 to ∼400 nm, allowing alternative excitation wavelengths to monitor oxygen effects. More details of the experimental setup are provided in the Experimental section. Figure 4 displays the recorded emission spectra normalized by the emission band relative to the Eu^III 5^D_0_ → ^7^F_2_ transition. It is important to clarify that performing measurements in suspension prevents direct comparison between the spectra in terms of absolute intensity, i.e. CPS, since the decantation of part of the particles influences the absolute intensity, Figure S23. Therefore, the spectra were normalized, avoiding problems related to variation in probe concentration.

**Figure 4:**
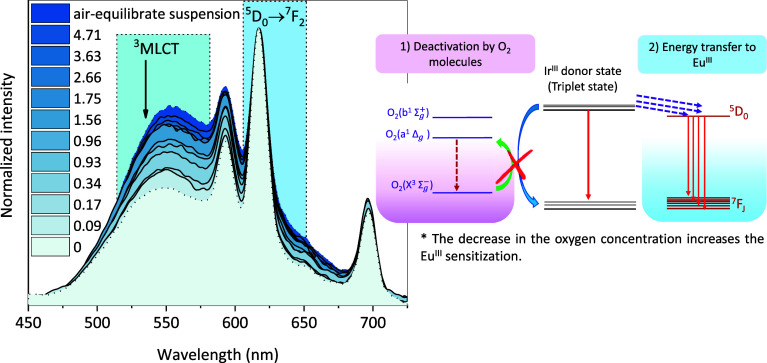
Emission spectra obtained at different dissolved oxygen (DO) concentrations, and schematic representation of two quenching channels of the Ir^III^-component. *The red “X” in the diagram on the right represents the decrease in oxygen content, which in turn acts increasing sensitization to Eu^III^. All measurements were carried out with a bandpass of 10 nm for both excitation and emission, with an increment of 0.5 nm and an integration time of 0.5 s λ_ex_ 250 nm.

Analyzing the normalized emission spectra, it is possible to see that the Ir^III^-moiety emission band decreases compared to Eu^III^ ion emission as the oxygen content decreases, indicating a more efficient sensitization process. The two main deactivation channels for Ir^III^ emission are also illustrated in Figure 4. The first one is the well-known and already mentioned oxygen quenching,^61^ and the second one is the energy transfer to the Eu^III^ ion.^62^ At high oxygen concentrations, the energy in the ^3^MLCT state is mainly quenched by oxygen molecules. As a result, the energy that would otherwise sensitize the Eu^III^ ion is lost through nonradiative pathways, reducing the sensitization efficiency. When oxygen molecules are removed, the energy in the ^3^MLCT state can be transferred to the emissive state of Eu^III^, enhancing the relative intensity of the red band compared to the green emission from Ir^III^ moiety. In summary, the decrease in the oxygen content increases the sensitization process to Eu^III^. Despite variations in the emission spectra, color perception remains nearly constant across different oxygen concentrations, as shown in the color diagram in Figure S24. The diagram indicates a shift toward red with increasing sensitization. However, analysis using MacAdam ellipses—which define regions where color differences are imperceptible—^63^ reveals that this probe does not function as a colorimetric sensor but can still be represented graphically.

To understand the increase in the sensitization process, lifetime measurements were performed before and after purging nitrogen gas in aqueous suspension. The emission lifetimes of the Ir^III^ moiety at 558 nm were obtained by multiexponential fitting and reported as average values (Figures S11 and S18). The presence of multiple lifetime components is expected, reflecting distinct microenvironments of the luminescent species on the silica surface arising from the limited control over the europium coordination number. These data showed that in degassed conditions, the average emission lifetime of the Ir^III^ moiety decreases from 21.2 to 15.3 ns, Table 2. M. Zeyrek Ongun and co-workers^64^ studied Ir^III^-based complexes to measure luminescence quenching caused by oxygen molecules. In their study, the emission lifetime of the ^3^MLCT state dropped from 54 to 64 μs to 10–11 μs when oxygen gas was purged in the complex solutions, the opposite behavior observed in the system under study here. The same behavior was reported by H. Liu and co-workers^46^ measuring dual-emission fluorescence and room-temperature phosphorescence for ratiometric and calorimetric oxygen sensing and detection based on the dispersion of pure organic thianthrene dimer in a polymer host. In both related systems, no secondary quenching pathway of the ^3^MLCT states is observed; however, in the present case, Eu^III^ ions act as energy acceptors. Consequently, photons that would otherwise be quenched by oxygen are transferred to the Eu^III^ emissive state. This behavior is supported by the measured ^5^D_0_ emission lifetime, which increases from 192.8 to 217.5 μs with decrease in the oxygen content, indicating enhanced Eu^III^ sensitization, as discussed previously and showed in Table 2.

Figure 5A exhibits a graphical representation of the luminescence ratio between Ir^III^ and Eu^III^ moieties emission dependent on the molecular oxygen concentration. Herein, it is possible to observe that with the decrease in oxygen concentration, the relative emission intensity of the Eu^III^ ion increases compared to Ir^III^ emission component. Therefore, it is possible to point out that the Ir^III^/Eu^III^ ratio has no linear dependence on the oxygen concentration.

Luminescent quenching can be related to different deactivation mechanisms, such as the formation of complexes between the luminophore and the quencher, known as static quenching, and/or collision, which is called dynamic quenching. Both require contact between the luminophores in their excited states and the quencher.^65^ Lifetime values were critical for understanding the quenching mechanism in this system. In dynamic quenching, the emission lifetime is affected, as well as the emission intensity because deactivation occurs after the collision of a molecule in the excited state and the quencher. In contrast, the deactivation process in static quenching affects the emission intensity but does not affect the lifetime of the probe because the excited state is generated after complex formation between the probe molecules and the analyte under study.^53,66^ In this second mechanism, the energy in the excited state immediately returns to the ground state without photon emission. In conclusion, it is possible to say that the main quenching mechanism in the system under study is the dynamic one since changes in the measured lifetime (from 21.2 to 15.3 ns) were observed.

To determine the sensitivity of a probe, the Stern–Volmer model is used, which states that the variation in emission intensity has a linear dependence on the concentration of the analyte. The Stern–Volmer equation correlates the emission lifetime (τ) or emission quantum yield (Φ) of a luminophore upon the addition of a quencher at different concentrations [Q], which is represented by eq 3

3 $$\frac{\Phi_{0}}{\Phi }=\frac{\tau_{0}}{\tau }=1+K_{q}\tau_{0}\left[Q\right]$$

in this equation, the Φ_0_ and τ_0_ represent the respective values at [*Q*] = 0, that is in the absence of the quencher, thereby Φ and τ are given upon quencher addition. *K*_*q*_ is the bimolecular rate constant of the luminescence quenching process due to the short-range interaction of species. If the emission quantum yield remains proportional to the integrated emission area upon quencher addition, eq 2 can be simplified by using the luminescence intensity ratio, as represented by eq 4

4 $$\frac{I_{0}}{I}=1+K_{SV}\left[Q\right]$$

where *K*_SV_ (=*K*_*q*_τ_0_) is the Stern–Volmer constant. Considering a dynamic fluorescence quenching process, *K*_SV_ is the product of the bimolecular quenching rate constant and the fluorescence lifetime in the absence of the quencher, and it represents the Stern–Volmer dynamic quenching constant. The same equation can be used to understand static quenching, however, the *K*_SV_ represents the association constant between the luminophore and quencher. The plot between *I*_o_/*I* and [Q] can be found in Figure 5B). In this graph, *I*_o_ is the Ir^III^/Eu^III^ ratio in the absence of oxygen, [DO] = 0%, and the *I* value is the Ir^III^/Eu^III^ ratio at different oxygen concentration. Since the system under study no longer describes a linear dependence, we used the two-site Stern–Volmer model proposed by Demas and co-workers^67^ to understand a system with different emissive microzones. To fit the two-site model, the following equation was applied

5 $$\frac{I_{0}}{I}=\frac{\tau_{0}}{\tau }=\frac{1}{\frac{f_{a}}{1+K_{SV}^{a}\left[O_{2}\right]}+\frac{f_{b}}{1+K_{SV}^{b}\left[O_{2}\right]}}$$

where *K*_SV_^*a*^ and *K*_SV_^*b*^ are the Stern–Volmer constants of different microzone; and *f*_*a*_ and *f*_*b*_ are their respective component proportions, in which *f*_*a*_ + *f*_*b*_ equals 1. The following parameters were obtained: *f*_*a*_ = 0.47, *f*_*b*_ = 0.53, *K*sv^a^ = 11.772 ppm^–1^, *K*sv^b^ = 0.026 ppm^–1^. The fact that *f*_*a*_ and *f*_*b*_ are almost the same, close to 0.5, indicates a huge heterogeneity of the emissive sites formed on the silica surface; however, because the SV constants are very distinct, they are quenched at a very different rate.^68^ The high *K*_SV_^a^ value calculated for this system can be attributed to the high permeability of oxygen molecules on the silica surface to access the Ir^III^-component.

**Figure 5:**
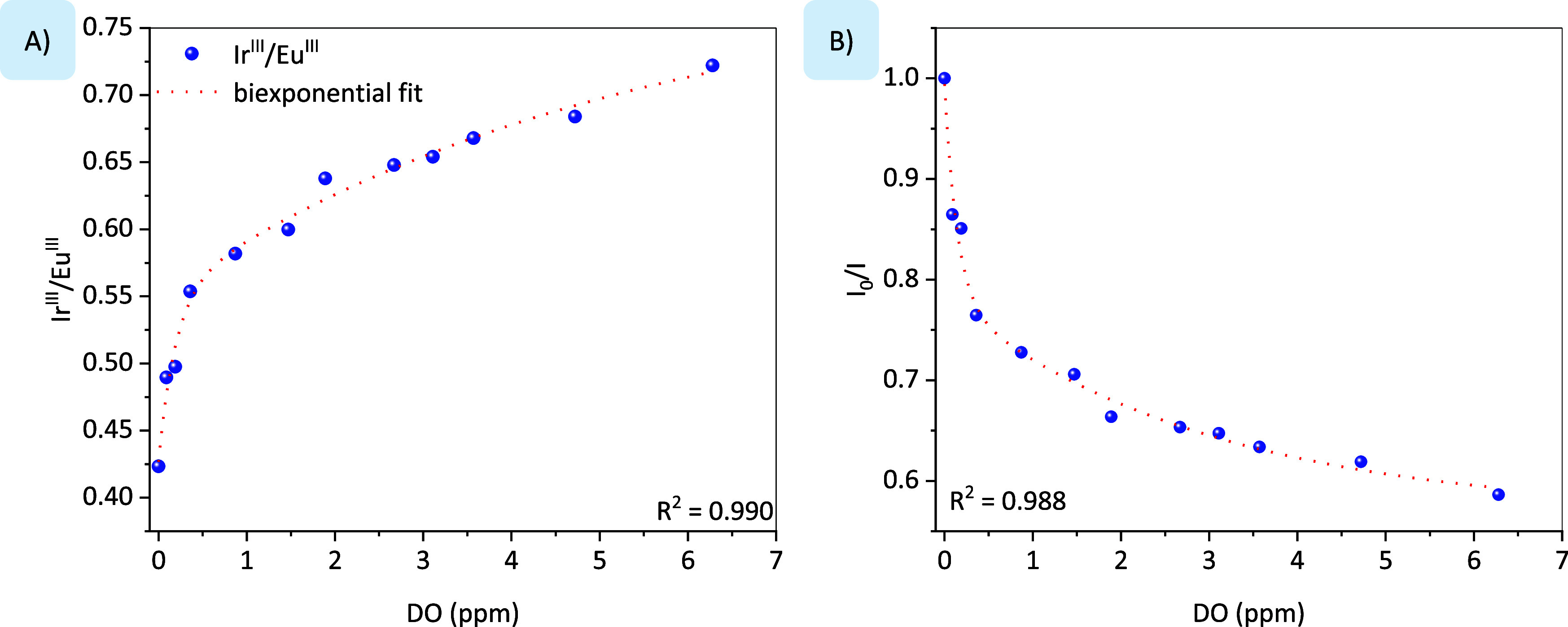
(A) Graphical representation of the Ir^III^/Eu^III^ ratio versus dissolved oxygen (DO) content, and (B) Stern–Volmer plot where *I*_0_ is the Ir^III^/Eu^III^ ratio with 0 ppm DO, and *I* is the Ir^III^/Eu^III^ ratio at different DO concentration.

The limit of detection (LOD) of each site can be estimated using the quenching plot slope (*K*_SV_) and the equipment resolution. LOD values were defined considering the DO concentration at which the sensor signal level reaches at least 3 times the baseline signal-to-noise ratio.^51^ Assuming the uncertainty of the measurement as 0.1%, the LOD (=0.003/*K*_sv_, where *K*_sv_ is expressed in ppm^–1^) were determined to be 0.00025 ppm and 0.1153 ppm, considering *K*_sv_^*a*^ and *K*_sv_^*b*^, respectively. Another important parameter is the oxygen sensitivity of the probe, which expresses the total spectral modification of the probe when the emission spectrum is acquired at air-equilibrated and at 0% of dissolved oxygen. This parameter can be expressed by the *Q* factor, which is calculated using the following equation |*Q*| = [(*I*_0_-*I*_air_)/*I*_0_]×100%, here I_o_ is the Ir^III^/Eu^III^ ratio in the absence of oxygen, and *I*_air_ is the ratio under air-equilibrated conditions. Here, the *Q* factor was calculated to be 70.54%, lower than values already reported in the literature for molecular probes.^69,70^ Considering that the system reported here relies on energy transfer between the Eu^III^ center and the Ir^III^-moiety, modifications in the electronic structure of the coordination environment may enhance the efficiency of this process and consequently improve oxygen sensitivity. Structural variations in the cyclometalated and ancillary (bridging) ligands can modulate the emission profile of the Ir^III^-based unit, while the precise energetic alignment between the triplet donor state of the Ir^III^ center and the acceptor Eu^III^ (^5^D_0_) excited state is crucial for maximizing the energy-transfer efficiency and, therefore, the overall sensitivity of the probe.

### Cytotoxic Study

The cytotoxic assays were performed in the Huh 7.5 cell line (human hepatocellular carcinoma cell line) by the MTT 3-(4,5-dimethylthiazol-2-yl)-2,5-diphenyltetrazolium bromide) reduction method to analyze the particle toxicity in the biological medium of the final luminescent hybrid, SiO_2_–Eu^III^Ir^III^. After 24 h of exposure, the final hybrid was considered nontoxic at all concentrations tested according to the international standard ISO 10993-5:2009 (Biological evaluation of medical devices—Part 5: Tests for *in vitro* cytotoxicity),^71^ which claims that the tested material is nontoxic when cell viability is equal to or greater than 70%. The cell viability obtained by varying the material concentration is shown in Figure 6. Wu et al.^72^ and Zhao et al.,^73^ in two different studies, reported that mesoporous silica particles decorated with Eu^III^ complexes were nontoxic in concentrations between 25 and 200 μg mL^–1^ when tested in Hella cells after 24 h of exposure; in our report, on the other hand, the nontoxic concentration extends to the limit of analysis, 400 μg mL^–1^. Yu et al.^74^ reported that the main factor that influences the toxicity of amorphous silica in biological systems is the size of the particles; normally, particles smaller than 100 nm are more toxic. In addition, Al-Rawi et al.^75^ observed that silica particles with size between 200 and 500 nm are much less toxic than particles with 70 nm. Thereby, it is comprehensive that the silica particles, here under study, are classified as nontoxic because their average size is 288.8 ± 24.9 nm, as determined previously by the TEM technique. The larger the particle size, the smaller the surface area exposed to the cellular environment, leading to a lower formation of reactive oxygen species (ROS) that cause cell death. Although size is an important requirement for toxicity, it is not the only one; i.e. surface potential can also affect the way nanoparticles interact with cells.^76^ In our research group, we have shown that when nanoparticles are suspended in a DMEM culture medium, their surface potential, given by the zeta potential, becomes weakly negative around −12 mV due to the effect of the protein corona.^77^ Drawing a comparison with the present study, the weakly negative surface potential and the possibility of the corona effect may be positive points for an efficient cellular uptake despite the large size of the particles.

**Figure 6:**
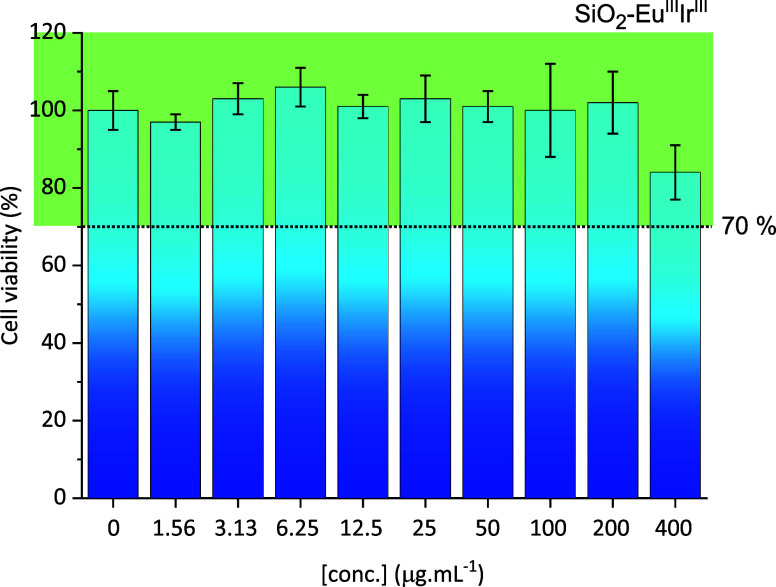
Cell viability of SiO_2_–Eu^III^Ir^III^ sample obtained from the Huh 7.5 cell line using MTT assays.

### Cell Imaging Study

To attest the potential applicability of the final luminescent hybrid as a cell image agent, SiO_2_–Eu^III^Ir^III^, a suspension with a concentration considered nontoxic, 50 μg mL^–1^, was prepared, and Huh 7.5 cells were then exposed to this suspension for 2 h. To delimit the nuclear region, Hoechst dye^78^ was used, which is a specific nuclear dye, as shown in Figure 7A. Thus, when compared with other images, it is possible to analyze whether the luminescent silica particles are internalizing or not. To obtain the images related to the Hoechst dye, an excitation laser at 405 nm was used, and the emission of the biomaterial was read on the DAPI channel, in the blue spectral region. To certify the luminescent profile of the Ir^III^ and Eu^III^ moieties, confocal images were obtained using the same excitation source, a laser at 488 nm. To analyze the emission of the Ir^III^ moiety, the data were read on the FTIC channel, in the green spectral region. Figure 7B shows the emission of the Ir^III^ moiety in silica particles internalized by cell membranes, and in Figure 7E that there is no colocalization of the blue and green emission, by merging Figure 7A,B). Through these images, it is possible to state that the green emission of Ir^III^ comes from the cytoplasmic region, and additionally to infer that the silica particles do not cross the nuclear membrane because no green emission was detected in the nuclear region. Eu^III^ emission was read on the TEXAS RED channel, in the red spectral region. In Figure 7C) the red emission of the Eu^III^ ion is shown, and in Figure 7F) the merge between Figure 7C,A) can be seen. Again, it is possible to observe that the emission from the silica particles is placed around the nuclei in the cytoplasmic region. However, a merge between Figure 7B,C), gives rise to Figure 7D) that shows a perfect colocalization between the Ir^III^ and Eu^III^ emissions, as it was expected since they form a single platform, resulting in a final yellow color due to color mixing.

**Figure 7:**
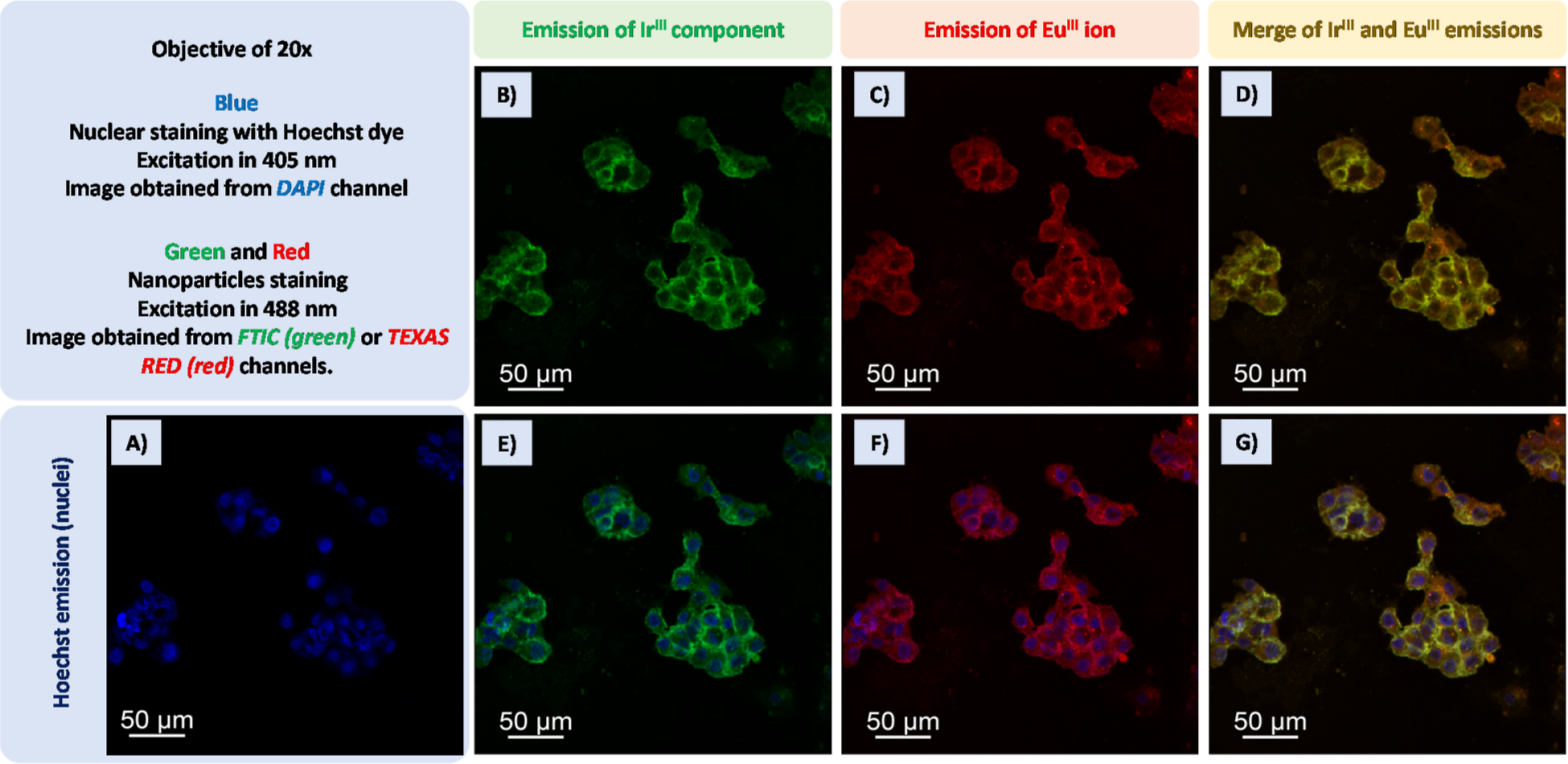
Confocal images of Huh cells incubated with SiO_2_–Eu^III^Ir^III^ sample (50 μg mL^–1^). (A) Nuclei stained with Hoechst dye; (B) Ir^III^ component emission; (C) Eu^III^ emission; (D) merge of B and C images; (E) merge of A and B images; (F) merge of A and C images; and (G) merge of A, B and C images.

Finally, Figure 7G) reveals the merge between the emission of Ir^III^ and Eu^III^ and the blue emission of the Hoechst dye. In a previous study reported by our research group,^42^ the internalization of functionalized silica particles with an average diameter of ∼120 nm was found to occur predominantly via an endocytic transport mechanism. Following membrane invagination, the particles are encapsulated within endosomal vesicles and subsequently distributed throughout the cytoplasm, eventually reaching the perinuclear region. Several studies in the literature have investigated the cellular uptake of inorganic nanoparticles and generally report two main mechanisms: (i) phagocytosis, typically associated with particles larger than 500 nm, and (ii) endocytosis, which predominates for particles smaller than 500 nm.^79^ Kim et al.^80^ investigated the uptake of silica particles with different sizes and concluded that endocytosis is the dominant pathway, although some particles were also detected free within the cytosol and mitochondria. Similarly, Nabeshi et al.^81^ studied the intracellular distribution of silica nanoparticles in HaCaT cells and observed that smaller particles (∼70 nm) are widely distributed in the cytoplasm and can even penetrate the nucleus, whereas larger particles (300 and 1000 nm) are mainly confined within endosomal compartments. Thus, consistent with our previous report and literature findings, the functionalized silica particles introduced here are internalized by Huh cells by endocytosis process and distributed throughout the cytoplasm.

## Conclusions

Herein, the synthesis of silica particles decorated with Ir^III^–Eu^III^ heterobimetallic complex for oxygen sensing and cell labeling study was introduced. Morphological characterizations confirmed the formation of spherical particles with an average size of 288.8 ± 24.9 nm. The functionalization process was attested by elemental and thermal analysis, and by zeta potential, exhibiting a potential value of −14.20 mV, which is adequate to form a stable colloidal suspension. From the photoluminescent characterization, the dual-emission character of the material was evidenced, with the broad green emission band coming from the Ir^III^ moiety, and the narrow red emission bands from the Eu^III^, yielding a yellow overall emission. The excitation band of the final hybrid extends from 250 nm to approximately 550 nm, which is a crucial feature when bio application is intended because low excitation energy can be used to excite the luminescent probe, avoiding possible damage to biological tissues and autofluorescence process. Oxygen sensing measurements revealed that the hybrid has a nonlinear sensing response to oxygen concentration. Using the two-site Ster-Volmer model, it was observed to have an almost equal frequency of the two sites. However, the quenching constants determined exhibited very different values, evidencing distinct sensitivities to oxygen molecules. With the decrease in oxygen content, the Ir^III^ emission moiety exhibits a decrease in the emission intensity compared to the Eu^III^ ion. Decreasing the oxygen content increases the Eu^III^ ion sensitization process, which can be evidenced by the decrease in the emission lifetime of the ^3^MLCT state and by the increase in the Eu^III^ emission lifetime. In addition, by the lifetime, it was possible to conclude that the main quenching mechanism in this system is the dynamic one with an oxygen sensitivity of 70.5%. Finally, toxicity assays were performed using the Huh-7.5 cell line, and the SiO_2_–Eu^III^Ir^III^ particles were nontoxic in concentrations between 1.56 and 400 μg mL^–1^, and confocal analyses proved that the particles were internalized by the cells, maintaining their luminescent properties because green and red emissions were separately detected using the same excitation laser. These results ensure that this material is a promising ratiometric luminescent probe for labeling cells and detecting oxygen in biological medium.

## Supplementary Material



## Notes

Characterization and data analysis procedures (PDF)

## References

[1] Aragonés J., Fraisl P., Baes M., Carmeliet P. (2009). Oxygen sensors
at the crossroad of metabolism. Cell Metab..

[2] Kumari R., Sunil D., Ningthoujam R. S. (2019). Naphthalimides
in fluorescent imaging
of tumor hypoxia–An up-to-date review. Bioorg. Chem..

[3] Wen Y., Zhang S., Yuan W., Feng W., Li F. (2023). Afterglow/fluorescence
dual-emissive ratiometric oxygen probe for tumor hypoxia imaging. Anal. Chem..

[4] Salvato I., Marchini A. (2024). Immunotherapeutic strategies for
the treatment of glioblastoma:
current challenges and future perspectives. Cancers.

[5] Lawrence S. R., Shah K. M. (2024). Prospects and Current
Challenges of Extracellular Vesicle-Based
Biomarkers in Cancer. Biology.

[6] Ratcliffe P. J. (2013). Oxygen
sensing and hypoxia signalling pathways in animals: the implications
of physiology for cancer. J. Physiol..

[7] Passaro A., Al Bakir M., Hamilton E. G., Diehn M., André F., Roy-Chowdhuri S., Mountzios G., Wistuba I. I., Swanton C., Peters S. (2024). Cancer biomarkers:
emerging trends and clinical implications
for personalized treatment. Cell.

[8] Reese K. L., Pantel K., Smit D. J. (2024). Multibiomarker
panels in liquid biopsy
for early detection of pancreatic cancer–a comprehensive review. J. Exp. Clin. Cancer Res..

[9] Papkovsky D. B., Dmitriev R. I. (2013). Biological detection by optical oxygen
sensing. Chem. Soc. Rev..

[10] Tobita S., Yoshihara T. (2016). Intracellular and in vivo oxygen sensing using phosphorescent
iridium (III) complexes. Curr. Opin. Chem. Biol..

[11] Marassi V., Giordani S., Kurevija A., Panetta E., Roda B., Zhang N., Azzolini A., Dolzani S., Manko D., Reschiglian P. (2022). The Challenges of O2 Detection in Biological
Fluids: Classical Methods and Translation to Clinical Applications. Int. J. Mol. Sci..

[12] Joseph S., Kumar S. A. (2023). Recent advances in the photophysical detection and
delivery of singlet oxygen. Coord. Chem. Rev..

[13] Mrakic-Sposta S., Gussoni M., Montorsi M., Porcelli S., Vezzoli A. (2014). A quantitative
method to monitor reactive oxygen species production by electron paramagnetic
resonance in physiological and pathological conditions. Oxid. Med. Cell. Longevity.

[14] Oja J. M., Gillen J. S., Kauppinen R. A., Kraut M., Van Zijl P. C. (1999). Determination
of oxygen extraction ratios by magnetic resonance imaging. J. Cereb. Blood Flow Metab..

[15] Valable S., Corroyer-Dulmont A., Chakhoyan A., Durand L., Toutain J., Divoux D., Barré L., MacKenzie E. T., Petit E., Bernaudin M. (2017). Imaging of brain oxygenation
with magnetic resonance imaging: A validation with positron emission
tomography in the healthy and tumoural brain. J. Cereb. Blood Flow Metab..

[16] Nitzan M., Romem A., Koppel R. (2014). Pulse oximetry: fundamentals
and
technology update. Med. Devices: Evidence Res..

[17] Yoshihara T., Matsumura N., Tamura T., Shiozaki S., Tobita S. (2022). Intracellular
and intravascular oxygen sensing of pancreatic tissues based on phosphorescence
lifetime imaging microscopy using lipophilic and hydrophilic iridium
(III) complexes. ACS Sens..

[18] Phillips K. A., Stonelake T. M., Chen K., Hou Y., Zhao J., Coles S. J., Horton P. N., Keane S. J., Stokes E. C., Fallis I. A. (2018). Ligand-tuneable, red-emitting iridium (III)
complexes for efficient triplet–triplet annihilation upconversion
performance. Chem. Eur J..

[19] Brito, H. F. ; Malta, O. M. L. ; Felinto, M. C. F. C. ; Teotonio, E. E. de S. Luminescence Phenomena Involving Metal Enolates. In The Chemistry of Metal Enolates; Zabicky, J. , Ed.; Wiley: Chichester, 2009; pp 131–184. 10.1002/aoc.1628.

[20] Imran M., Chen M. S. (2022). Self-sensitized
and reversible O2 reactivity with bisphenalenyls
for simple, tunable, and multicycle colorimetric oxygen-sensing films. ACS Appl. Mater. Interfaces.

[21] Wu Y., Sutton G. D., Halamicek M. D., Xing X., Bao J., Teets T. S. (2022). Cyclometalated iridium-coumarin ratiometric oxygen
sensors: improved signal resolution and tunable dynamic ranges. Chem. Sci..

[22] Nam J. S., Kang M. G., Kang J., Park S. Y., Lee S. J. C., Kim H. T., Seo J. K., Kwon O.-H., Lim M. H., Rhee H.-W., Kwon T. H. (2016). Endoplasmic reticulum-localized iridium
(III) complexes as efficient photodynamic therapy agents via protein
modifications. J. Am. Chem. Soc..

[23] O’Donovan C., Hynes J., Yashunski D., Papkovsky D. B. (2005). Phosphorescent
oxygen-sensitive materials for biological applications. J. Mater. Chem..

[24] Abbas S., Din I. U. D., Raheel A., Tameez ud Din A. (2020). Cyclometalated
Iridium (III) complexes: Recent advances in phosphorescence bioimaging
and sensing applications. Appl. Organomet. Chem..

[25] Yu H., Yu B., Song Y., Hai P. (2023). Recent advances of cyclometalated
Ir (III) complexes for optical oxygen sensing. Inorg. Chim. Acta.

[26] Feng Y., Cheng J., Zhou L., Zhou X., Xiang H. (2012). Ratiometric
optical oxygen sensing: a review in respect of material design. Analyst.

[27] Grasso G., Onesto V., Forciniti S., D’Amone E., Colella F., Pierantoni L., Famà V., Gigli G., Reis R. L., Oliveira J. M. (2024). Highly
sensitive ratiometric fluorescent fiber matrices for oxygen sensing
with micrometer spatial resolution. Bio-Des.
Manuf..

[28] Pei X., Pan Y., Zhang L., Lv Y. (2021). Recent advances in ratiometric luminescence
sensors. Appl. Spectrosc. Rev..

[29] Sutton G. D., Jiang C., Liu G., Teets T. S. (2023). Ratiometric oxygen
sensors of cyclometalated iridium (III) with enhanced quantum yields
and variable dynamic ranges. Dalton Trans..

[30] Bigdeli A., Ghasemi F., Abbasi-Moayed S., Shahrajabian M., Fahimi-Kashani N., Jafarinejad S., Farahmand Nejad M. A., Hormozi-Nezhad M. R. (2019). Ratiometric fluorescent nanoprobes
for visual detection:
Design principles and recent advances-A review. Anal. Chim. Acta.

[31] Zhao Y., Li D. (2020). Lanthanide-functionalized
metal–organic frameworks as ratiometric
luminescent sensors. J. Mater. Chem. C.

[32] Miao W. N., Liu B., Li H., Zheng S. J., Jiao H., Xu L. (2022). Fluorescent
Eu3+/Tb3+ metal–organic frameworks for ratiometric temperature
sensing regulated by ligand energy. Inorg. Chem..

[33] Rzepiela J., Zychowicz M., Zakrzewski J. J., Ohkoshi S. I., Baś S., Chorazy S. (2026). Construction of ratiometric optical thermometers by
linking chiral cyclometalated dicyanidoiridates (III) with europium
(III) luminophores into cluster compounds. J.
Mater. Chem. C.

[34] Metherell A. J., Curty C., Zaugg A., Saad S. T., Dennison G. H., Ward M. D. (2016). Converting an intensity-based
sensor to a ratiometric
sensor: luminescence colour switching of an Ir/Eu dyad upon binding
of a V-series chemical warfare agent simulant. J. Mater. Chem. C.

[35] Zhang H., Jiang J., Gao P., Yang T., Zhang K. Y., Chen Z., Liu S., Huang W., Zhao Q. (2018). Dual-emissive
phosphorescent polymer probe for accurate temperature sensing in living
cells and zebrafish using ratiometric and phosphorescence lifetime
imaging microscopy. ACS Appl. Mater. Interfaces.

[36] Lin R. B., Liu S. Y., Ye J. W., Li X. Y., Zhang J. P. (2016). Photoluminescent
metal–organic frameworks for gas sensing. Adv. Sci..

[37] Dong R., Shen Z., Li H., Cheng J., Fu Y. (2024). Research progress
in fluorescent gas sensors based on MOFs. J.
Mater. Chem. C.

[38] Hussein H. A., Nazir M. S., Azra N., Qamar Z., Seeni A., Tengku Din T. A. D. A. A., Abdullah M. A. (2022). Novel drug and gene
delivery system and imaging agent based on marine diatom biosilica
nanoparticles. Mar. Drugs.

[39] Jaque D., Richard C., Viana B., Soga K., Liu X., García Solé J. (2016). Inorganic nanoparticles for optical
bioimaging. Adv. Opt. Photonics.

[40] Selvin P. R. (2002). Principles
and biophysical applications of lanthanide-based probes. Annu. Rev. Biophys. Biomol. Struct..

[41] Mutti A. M., Canisares F. S., Santos J. A., Santos B. C., Cavalcante D. G., Job A. E., Pires A. M., Lima S. A. (2023). Silica-based nanohybrids
containing europium complexes covalently grafted: structural, luminescent,
and cell labeling investigation. J. Sol-Gel
Sci. Technol..

[42] Canisares F. D. S. M., Silva R. C., Davolos M. R., Pires A. M., Lima S. A. M. (2023). Heterobimetallic
iridium III–europium III complex: the role of donor energy
on sensitising the Eu III ion. New J. Chem..

[43] Mosmann T. (1983). Rapid colorimetric
assay for cellular growth and survival: application to proliferation
and cytotoxicity assays. J. Immunol. Methods.

[44] Costa A. L., Pacheco C. J., Patiño J. C., Gomes A. M. d. S., Artizzu F., Lima S. A. M., Bispo-Jr A. G., Pires A. M. (2026). A Dual-Mode
Magnetic–Luminescent Hybrid: Fe3O4+ Y2O3: Yb3+, Tm3+ Decorated
with a Eu3+ β-Diketonate Complex. Mater.
Res. Bull..

[45] Rasband, W. ImageJ; Version 1.53e; Java 1.8.0_112 (64 Bit); National Institute of Health: Bethesda, MD, USA, Available online: https://imagej.net/Wayne_Rasband (accessed 7/4/2025).

[46] Mutti A. M.
G., Santos J. A. O., Cavalcante D. G. S. M., Gomes A. S., Job A. E., Pires A. M., Lima S. A. M. (2019). Decorated silica particles with terbium
complexes as luminescent biomarker for cell imaging. Opt. Mater..

[47] Mutti A. M. G., Santos J. A. O., Cavalcante D. G. S. M., Gomes A. S., Job A. E., Teixeira G. R., Pires A. M., Lima S. (2019). Design of a red-emitter
hybrid material for bioimaging: europium complexes grafted on silica
particles. Mater. Today Chem..

[48] Allen L. H., Matijevíc E., Meites L. (1971). Exchange of Na+ for
the silanolic
protons of silica. J. Inorg. Nucl. Chem..

[49] Pabisch S., Feichtenschlager B., Kickelbick G., Peterlik H. (2012). Effect of interparticle
interactions on size determination of zirconia and silica based systems–A
comparison of SAXS, DLS, BET, XRD and TEM. Chem.
Phys. Lett..

[50] Montes D., Henao J., Taborda E. A., Gallego J., Cortés F. B., Franco C. A. (2020). Effect of textural
properties and surface chemical
nature of silica nanoparticles from different silicon sources on the
viscosity reduction of heavy crude oil. ACS
Omega.

[51] Manimaran M., Norizan M. N., Kassim M. H. M., Adam M. R., Abdullah N., Norrrahim M. N. F. (2025). Critical review on the stability and thermal conductivity
of water-based hybrid nanofluids for heat transfer applications. RSC Adv..

[52] Septiadi W. N., Trisnadewi I. A. N. T., Putra N., Setyawan I. (2018). Synthesis of hybrid
nanofluid with two-step method. E3S Web of Conferences.

[53] Harun M. A., Sidik N. A. C., Rohaizan M. A. M. (2021). Experimental
investigation of stability
and thermal properties of nanocellulose-water nanofluid. IOP Conference Series: Materials Science and Engineering.

[54] Xian H. W., Sidik N. A. C., Saidur R. (2020). Impact of
different surfactants and
ultrasonication time on the stability and thermophysical properties
of hybrid nanofluids. Int. Commun. Heat Mass
Transfer.

[55] Carnall W. T., Fields P. R., Rajnak K. (1968). Electronic
energy levels of the trivalent
lanthanide aquo ions. IV. Eu3+. J. Chem. Phys..

[56] Shafikov M. Z., Zaytsev A. V., Kozhevnikov V. N., Czerwieniec R. (2023). Aligning π-Extended
π-Deficient Ligands to Afford Submicrosecond Phosphorescence
Radiative Decay Time of Mononuclear Ir (III) Complexes. Inorg. Chem..

[57] Liu H., Pan G., Yang Z., Wen Y., Zhang X., Zhang S. T., Li W., Yang B. (2022). Dual-emission of fluorescence
and room-temperature
phosphorescence for ratiometric and colorimetric oxygen sensing and
detection based on dispersion of pure organic thianthrene dimer in
polymer host. Adv. Opt. Mater..

[58] Zanoni K. P., Kariyazaki B. K., Ito A., Brennaman M. K., Meyer T. J., Murakami Iha N. Y. (2014). Blue-green iridium­(III) emitter and
comprehensive photophysical elucidation of heteroleptic cyclometalated
iridium­(III) complexes. Inorg. Chem..

[59] Supkowski R. M., Horrocks Jr W. D. (2002). On the determination of the number of water molecules,
q, coordinated to europium (III) ions in solution from luminescence
decay lifetimes. Inorg. Chim. Acta.

[60] Iman K., Shahid M. (2019). Life sensors: current advances in
oxygen sensing by
lanthanide complexes. New J. Chem..

[61] Choung K. S., Marroquin K., Teets T. S. (2019). Cyclometalated iridium–BODIPY
ratiometric O 2 sensors. Chem. Sci..

[62] Etchells I. M., Pfrunder M. C., Williams J. G., Moore E. G. (2019). Quantification of
energy transfer in bimetallic Pt (ii)–Ln (iii) complexes featuring
an N̂ Ĉ N-cyclometallating ligand. Dalton Trans..

[63] Wood, M. (2010). MacAdam ellipses. Out of the Wood, Mike Wood Consulting LLC.(retrieved on June 8, 2011). Retrieved from the internet: URL: (http://www.mikewoodconsulting.com/articles/Protocol%20Fall).

[64] Zeyrek
Ongun M., Sahin M., Akbal T., Avsar N., Karakas H., Ertekin K., Atilla D., İbişoğlu H., Topal S. Z. (2020). Synthesis, characterization and oxygen sensitivity
of cyclophosphazene equipped-iridium (III) complexes. Spectrochim. Acta, Part A.

[65] Gehlen M. H. (2020). The centenary
of the Stern-Volmer equation of fluorescence quenching: From the single
line plot to the SV quenching map. J. Photochem.
Photobiol., C.

[66] DeGraff, B. A. ; Demas, J. N. Luminescence-based oxygen sensors. In Reviews in fluorescence 2005; Springer: US, 2005; pp 125–151.

[67] Carraway E. R., Demas J. N., DeGraff B. A., Bacon J. R. (1991). Photophysics and
photochemistry of oxygen sensors based on luminescent transition-metal
complexes. Anal. Chem..

[68] Wang X. D., Wolfbeis O. S. (2014). Optical methods for sensing and imaging
oxygen: materials,
spectroscopies and applications. Chem. Soc.
Rev..

[69] Xu X. Y., Yan B. (2016). Nanoscale LnMOF-functionalized
nonwoven fibers protected by a polydimethylsiloxane
coating layer as a highly sensitive ratiometric oxygen sensor. J. Mater. Chem. C.

[70] Wang R. F., Peng H. Q., Chen P. Z., Niu L. Y., Gao J. F., Wu L. Z., Tung C., Chen Y., Yang Q. (2016). A Hydrogen-Bonded-Supramolecular-Polymer-Based
Nanoprobe for Ratiometric Oxygen Sensing in Living Cells. Adv. Funct. Mater..

[71] Standard, I. Biological evaluation of medical devices—Part 5: Tests for in vitro cytotoxicity; International Organization for Standardization: Geneve, Switzerland, 2009; Vol. 10, p 9781570203558.

[72] Wu Y., Shi M., Zhao L., Feng W., Li F., Huang C. (2014). Visible-light-excited
and europium-emissive nanoparticles for highly-luminescent bioimaging
in vivo. Biomaterials.

[73] Zhao Q., Liu Y., Cao Y., Lv W., Yu Q., Liu S., Liu X., Shi M., Huang W. (2015). Rational Design of Nanoparticles
with Efficient Lanthanide Luminescence Sensitized by Iridium (III)
Complex for Time-Gated Luminescence Bioimaging. Adv. Opt. Mater..

[74] Yu K. O., Grabinski C. M., Schrand A. M., Murdock R. C., Wang W., Gu B., Schlager J. J., Hussain S. M. (2009). Toxicity of amorphous silica nanoparticles
in mouse keratinocytes. J. Nanopart. Res..

[75] Al-Rawi M., Diabaté S., Weiss C. (2011). Uptake and intracellular localization
of submicron and nano-sized SiO 2 particles in HeLa cells. Arch. Toxicol..

[76] Forest V., Pourchez J. (2017). Preferential binding
of positive nanoparticles on cell
membranes is due to electrostatic interactions: A too simplistic explanation
that does not take into account the nanoparticle protein corona. Mater. Sci. Eng. C.

[77] Santos J. A. O., Brito L. D., da Costa P. I., Pires A. M., Lima S. A. M. (2023). Development
of red-luminescent hybrids as contrast agents for cell imaging: A
correlation among surface, luminescence, and biological properties. Opt. Mater..

[78] Bucevičius J., Lukinavičius G., Gerasimaitė R. (2018). The use of hoechst dyes for DNA staining
and beyond. Chemosensors.

[79] Xu Z. P., Zeng Q. H., Lu G. Q., Yu A. B. (2006). Inorganic
nanoparticles
as carriers for efficient cellular delivery. Chem. Eng. Sci..

[80] Kim I. Y., Joachim E., Choi H., Kim K. (2015). Toxicity of
silica
nanoparticles depends on size, dose, and cell type. Nanomed. Nanotechnol. Biol. Med..

[81] Nabeshi H., Yoshikawa T., Matsuyama K., Nakazato Y., Matsuo K., Arimori A., Isobe M., Tochigi S., Kondoh S., Hirai T. (2011). Systemic distribution, nuclear entry and cytotoxicity
of amorphous nanosilica following topical application. Biomaterials.

