# Supplementary material for: Nontoxic Dual-Function Probe for Ratiometric Oxygen Sensing and Cellular Imaging Based on IrIII–EuIII-Functionalized SiO2 Particles
Source: ACS Omega. 2026 May 21;11(21):30998–1012. doi: 10.1021/acsomega.6c00449 (PMC13234894; doi:10.1021/acsomega.6c00449)
Supplement: Supplementary file 1 [file ao6c00449_si_001.pdf]

Nontoxic dual-function probe for ratiometric oxygen sensing and cellular imaging based on Ir^{III}-Eu^{III}-functionalized SiO_2 particles

Felipe S. M. Canisares,[a,b] *Alessandra M. G. Mutti,*[c] *João Antonio O. Santos,*[a] *Alessandro B. S. Garcia,*[a] *Marian R. Davolos,*[a] *Ana M. Pires,*[a,c,d] *Sergio A. M. Lima*[a,c,d*]

[a] São Paulo State University (UNESP), Institute of Chemistry, Av. Prof. Francisco Degni, 55 - Jardim Quitandinha, 14800-900, Araraquara-SP, Brazil.

[b] University of São Paulo (USP), Institute of Chemistry, Av. Prof. Lineu Prestes, 748 - Butantã, 05508-900, São Paulo-SP, Brazil.

[c] São Paulo State University (UNESP), School of Technology and Sciences, R. Roberto Símonsen, 305 - Centro Educacional, 19060-900, Presidente Prudente-SP, Brazil.

[d] São Paulo State University (Unesp), Institute of Biosciences, Humanities and Exact Sciences, R. Cristóvão Colombo, 2265 - Jardim Nazareth, CEP 15054-000, São José do Rio Preto-SP, Brazil.

KEYWORDS: luminescent materials; multifunctional platform; heterobimetallic complex (Ir^{III}-Ln^{III}); ratiometric probe; oxygen sensing; cell imaging

Figure S1. Thermogravimetric curves of the silica samples. A) SiO_2, B) SiO_2-NCO, C) SiO_2-COOH, D) SiO_2-Eu^{III}, and E) SiO_2-$Eu^{III}Ir^{III}$. 4
Figure S2. FTIR- spectra of SiO_2 (black), SiO_2-NCO (red), SiO_2-COOH (blue), SiO_2-Eu^{III} (green), and SiO_2-$Eu^{III}Ir^{III}$ (purple), and Ir^{III}-p (yellow)....5
Figure S3. Experimental and theoretical ratios of C/N in the SiO_2-COOH, SiO_2-Eu^{III}, and SiO_2-$Eu^{III}Ir^{III}$ samples. *The degree of functionalization was determined using the carbon and nitrogen percentages of the SiO_2-COOH sample since only organic matter was grafted onto it. For the sake of clarity, there is only one Ir^{III} represented in this illustration, but for the estimation of the C/N ratio of SiO_2-$Eu^{III}Ir^{III}$, two Ir^{III} complexes coordinated to the Eu^{III} ion were considered....6
Figure S4. Surface charge estimated by zeta potential of the SiO_2, SiO_2-NCO, SiO_2-COOH, SiO_2-Eu^{III}, and SiO_2-$Eu^{III}Ir^{III}$ samples. 6
Figure S5. The stability index measurement for SiO_2-$Eu^{III}Ir^{III}$ (0.1 mg mL^{-1}) in aqueous and DMEM suspension. 7
Figure S6. Emission spectra of SiO_2, SiO_2-NCO, SiO_2-COOH, SiO_2-$Eu^{III,}$ and SiO_2-$Eu^{III}Ir^{III}$ measured in the solid state at room temperature. All measurements were carried out with a bandpass of 2.5 nm for both Ex and Em, with an increment of 0.5 nm and an integration time of 0.5 s. λ_{ex} 393 nm. 8
Figure S7. Excitation spectra of SiO_2, SiO_2-NCO, SiO_2-COOH, SiO_2-$Eu^{III,}$ and SiO_2-$Eu^{III}Ir^{III}$ measured in the solid state at room temperature. All measurements were carried out with a bandpass of 2.5 nm for both Ex and Em, with an increment of 0.5 nm and an integration time of 0.5 s. λ_{em} 617 nm. 8
Figure S8. Emission lifetime of Eu^{III} in SiO_2-Eu^{III} sample in powder, obtained upon excitation at 393 nm and emission at 617 nm....9
Figure S9. Emission lifetime of Eu^{III} in SiO_2-$Eu^{III}Ir^{III}$ sample in powder, obtained upon excitation at 393 nm and emission at 617 nm. 9
Figure S10. Emission lifetime of Eu^{III} in SiO_2-Eu^{III} sample in aqueous suspension, obtained upon excitation at 393 nm and emission at 617 nm. 10
Figure S11. Emission lifetime of Eu^{III} in SiO_2-$Eu^{III}Ir^{III}$ sample in aqueous suspension, obtained upon excitation at 393 nm and emission at 617 nm. 10
Figure S12. Emission lifetime of Ir^{III} component in SiO_2-$Eu^{III}Ir^{III}$ sample in aqueous suspension, obtained upon excitation at 375 nm and emission at 558 nm....11

Figure S13. Emission lifetime of Eu^{III} in SiO_2-Eu^{III} sample in deuterated aqueous suspension, obtained upon excitation at 393 nm and emission at 617 nm. 11
Figure S14. Emission lifetime of Eu^{III} in SiO_2-$Eu^{III}Ir^{III}$ sample in deuterated aqueous suspension, obtained upon excitation at 393 nm and emission at 617 nm. 12
Figure S15. Emission lifetime of Ir^{III} component in SiO_2-$Eu^{III}Ir^{III}$ sample in deuterated aqueous suspension, obtained upon excitation at 375 nm and emission at 558 nm. 12
Figure S16. Emission lifetime of Eu^{III} in SiO_2-Eu^{III} sample in DMEM suspension, obtained upon excitation at 393 nm and emission at 617 nm. 13
Figure S17. Emission lifetime of Ir^{III} component in SiO_2-$Eu^{III}Ir^{III}$ sample in DMEM suspension, obtained upon excitation at 375 nm and emission at 558 nm. 13
Figure S18. Emission lifetime of Eu^{III} in SiO_2-Eu^{III} sample in degassed aqueous suspension, obtained upon excitation at 393 nm and emission at 617 nm. 14
Figure S19. Emission lifetime of Ir^{III} component in SiO_2-$Eu^{III}Ir^{III}$ sample in degassed aqueous suspension, obtained upon excitation at 375 nm and emission at 558 nm. 14
Figure S20. Excitation spectra of SiO_2-Eu^{III} and SiO_2-$Eu^{III}Ir^{III}$ samples measured in aqueous and deuterated aqueous suspension. (λ_{em} 617 nm) 15
Figure S21. Emission spectra of SiO_2-Eu^{III} and SiO_2-$Eu^{III}Ir^{III}$ samples measured in aqueous and deuterated aqueous suspension. (λ_{ex} 393 nm) 15
Figure S22. Excitation and Emission spectra of SiO_2-$Eu^{III}Ir^{III}$ samples measured in DMEM suspension. (λ_{em} 617 nm and λ_{ex} 393 nm) 16
Figure S23. Raw emission spectra obtained at different dissolved oxygen (DO) concentrations. 16
Figure S24. Variation in the color perception represented by CIE 1931 2° color coordinates. 17

SUPPLEMENTARY INFORMATION – SECTION I

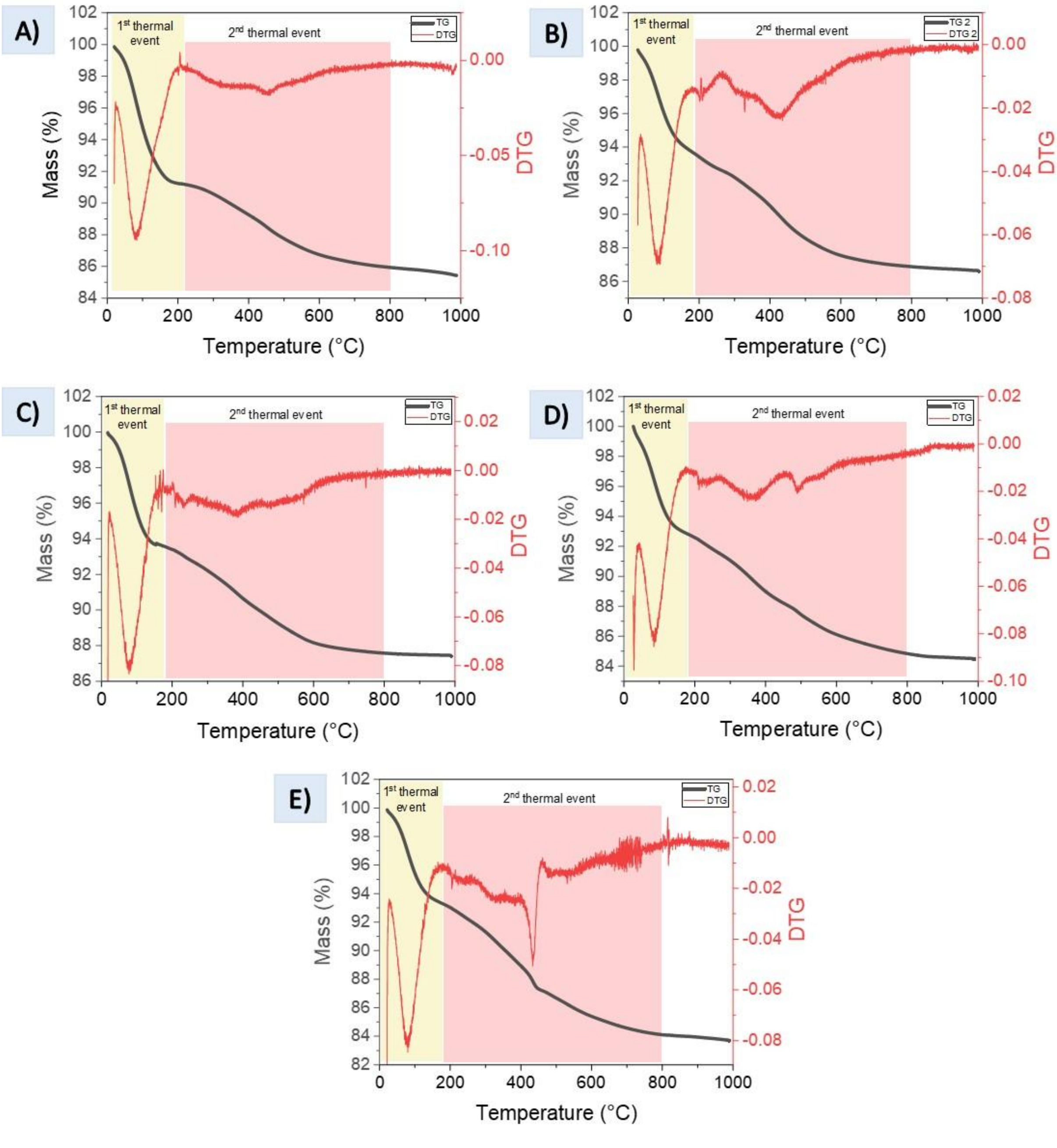

Figure S1. Thermogravimetric curves of the silica samples. A) SiO_2, B) SiO_2-NCO, C) SiO_2-COOH, D) SiO_2-Eu^{III}, and E) SiO_2-$Eu^{III}Ir^{III}$.

Table S1. Mass loss for each thermal event is evidenced by thermogravimetric (TGA) curves.

Samples	Weight loss (in %) observed in the 1st thermal event (up to ~200°C)	Weight loss (in %) observed in the 2nd thermal event (~200-800°C)
**SiO_2**	8,65%	5,27%
**SiO_2-NCO**	6,11%	6,81%
**SiO_2-COOH**	6,33%	6,06%
**SiO_2-Eu^{III}**	7,13%	8,03%
**SiO_2-$Eu^{III}Ir^{III}$**	6,57%	9,20%

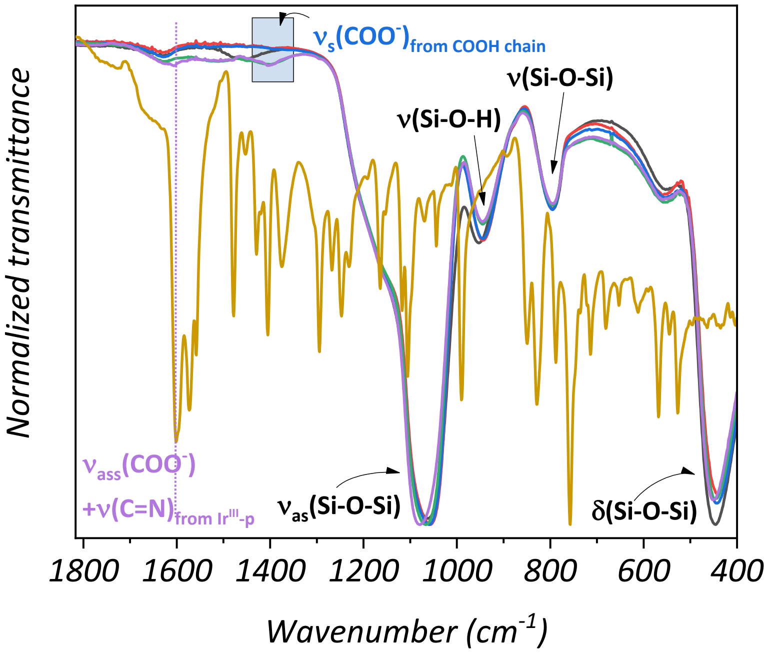

Figure S2. FTIR- spectra of SiO_2 (black), SiO_2-NCO (red), SiO_2-COOH (blue), SiO_2-Eu^{III} (green), and SiO_2-$Eu^{III}Ir^{III}$ (purple), and Ir^{III}-p (yellow).

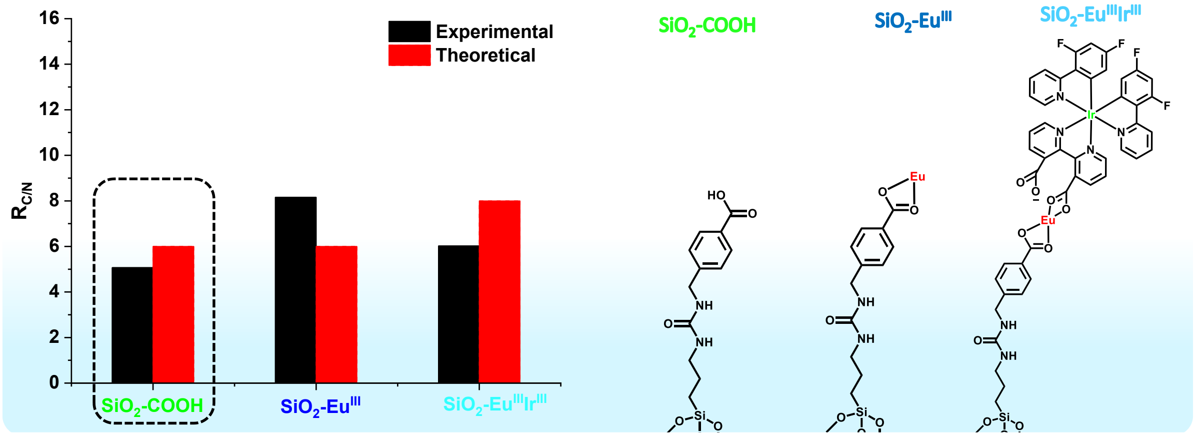

Figure S3. Experimental and theoretical ratios of C/N in the SiO_2-COOH, SiO_2-Eu^{III}, and SiO_2-$Eu^{III}Ir^{III}$ samples. *The degree of functionalization was determined using the carbon and nitrogen percentages of the SiO_2-COOH sample since only organic matter was grafted onto it. For the sake of clarity, there is only one Ir^{III} represented in this illustration, but for the estimation of the C/N ratio of SiO_2-$Eu^{III}Ir^{III}$, two Ir^{III} complexes coordinated to the Eu^{III} ion were considered.

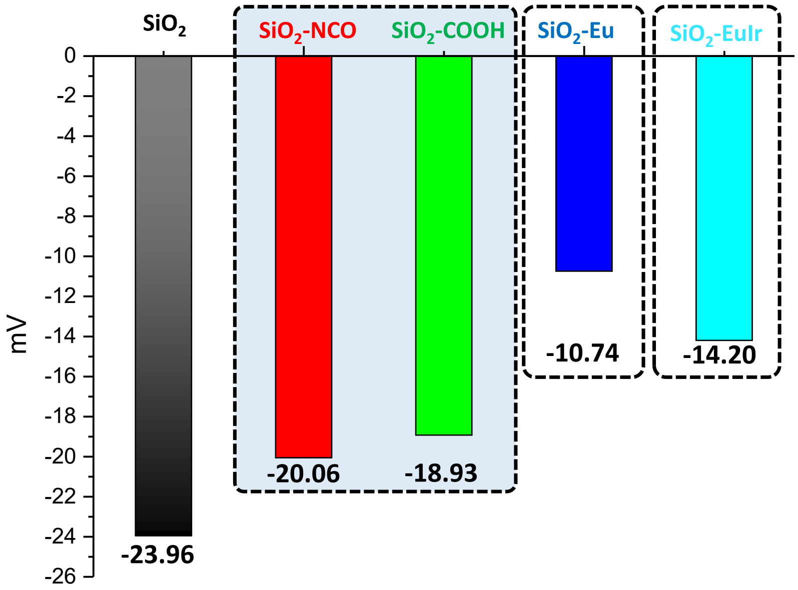

Figure S4. Surface charge estimated by zeta potential of the SiO_2, SiO_2-NCO, SiO_2-COOH, SiO_2-Eu^{III}, and SiO_2-$Eu^{III}Ir^{III}$ samples.

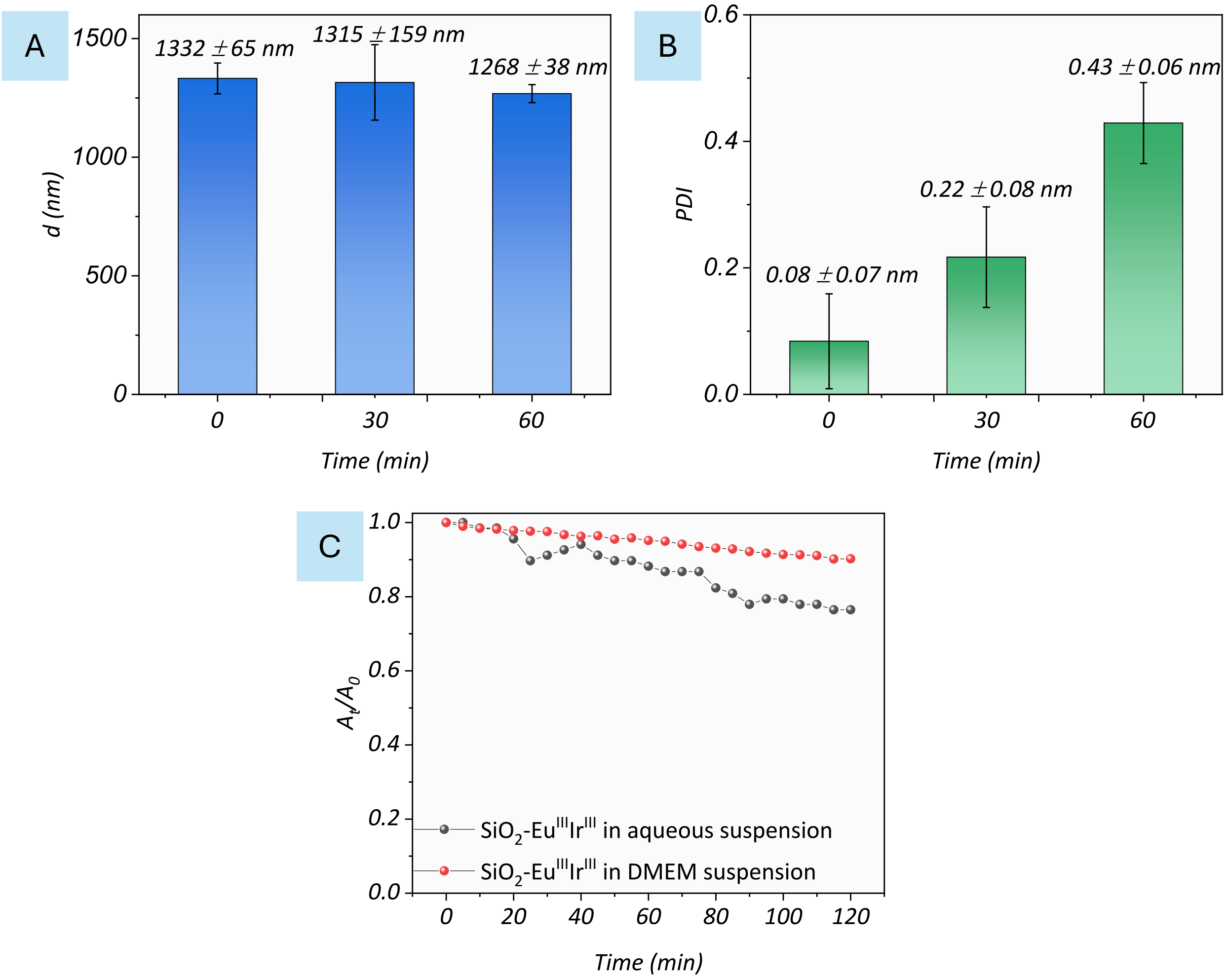

Figure S5. A) Hydrodynamic diameter, and B) Polydispersity index measured by DLS for diluted solutions; C) The stability index measurement for SiO_2-$Eu^{III}Ir^{III}$ (0.1 mg mL^{-1}) in aqueous and DMEM suspension.

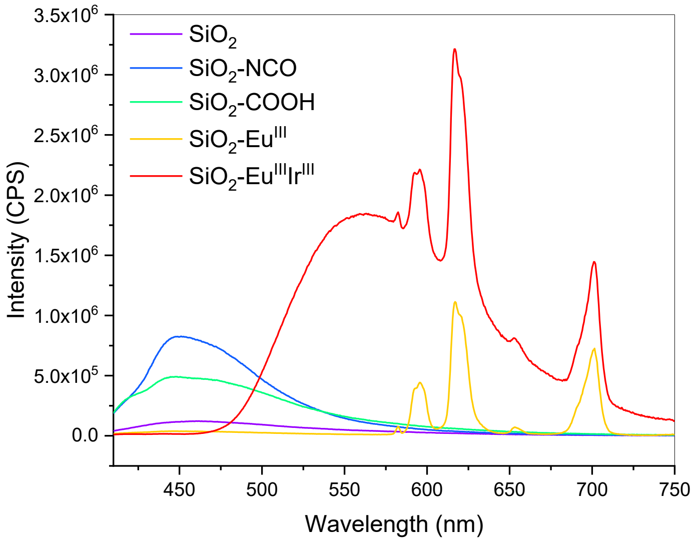

Figure S6. Emission spectra of SiO_2, SiO_2-NCO, SiO_2-COOH, SiO_2-$Eu^{III,}$ and SiO_2-$Eu^{III}Ir^{III}$ measured in the solid state at room temperature. All measurements were carried out with a bandpass of 2.5 nm for both Ex and Em, with an increment of 0.5 nm and an integration time of 0.5 s. λ_{ex} 393 nm.

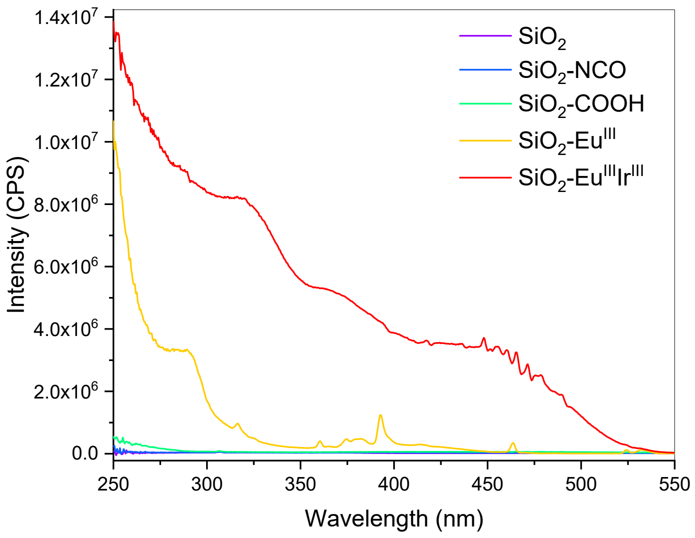

Figure S7. Excitation spectra of SiO_2, SiO_2-NCO, SiO_2-COOH, SiO_2-$Eu^{III,}$ and SiO_2-$Eu^{III}Ir^{III}$ measured in the solid state at room temperature. All measurements were carried out with a bandpass of 2.5 nm for both Ex and Em, with an increment of 0.5 nm and an integration time of 0.5 s. λ_{em} 617 nm.

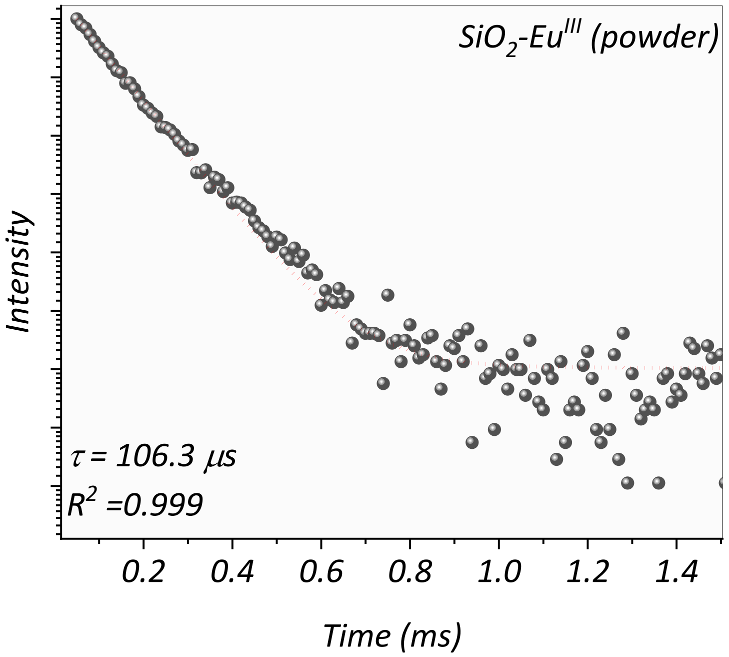

Figure S8. Emission lifetime of Eu^{III} in SiO_2-Eu^{III} sample in powder, obtained upon excitation at 393 nm and emission at 617 nm.

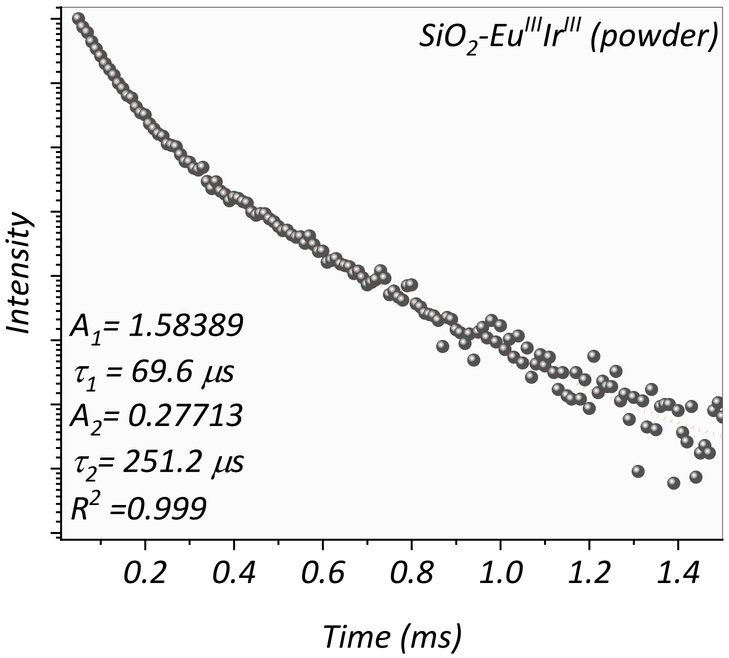

Figure S9. Emission lifetime of Eu^{III} in SiO_2-$Eu^{III}Ir^{III}$ sample in powder, obtained upon excitation at 393 nm and emission at 617 nm.

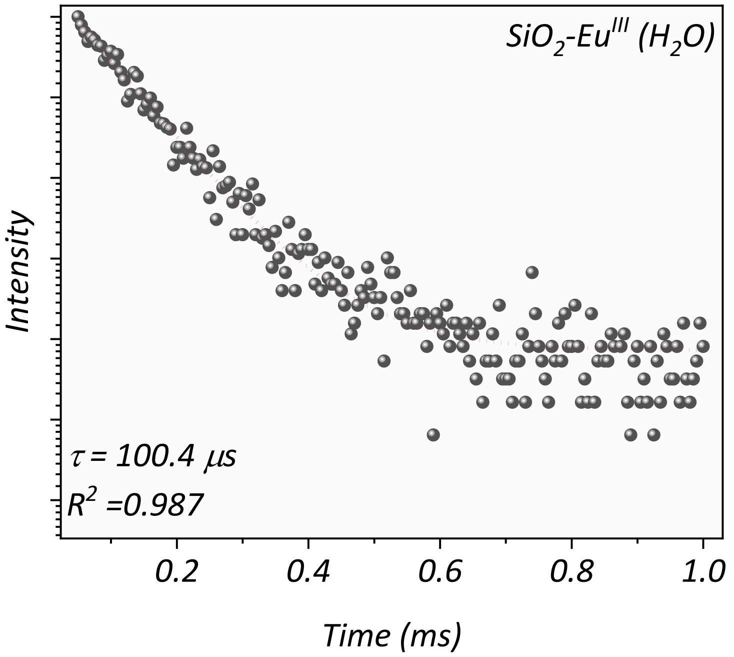

Figure S10. Emission lifetime of Eu^{III} in SiO_2-Eu^{III} sample in aqueous suspension, obtained upon excitation at 393 nm and emission at 617 nm.

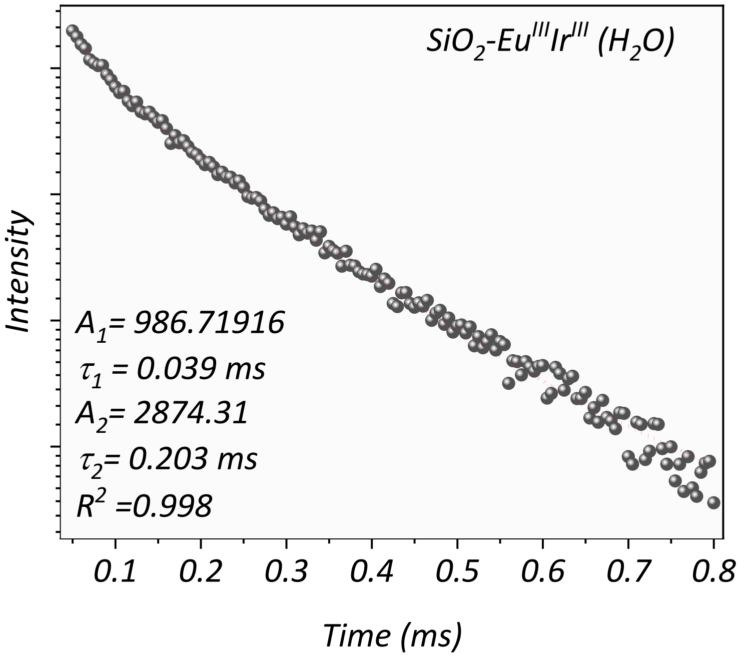

Figure S11. Emission lifetime of Eu^{III} in SiO_2-Eu^{III}Ir^{III} sample in aqueous suspension, obtained upon excitation at 393 nm and emission at 617 nm.

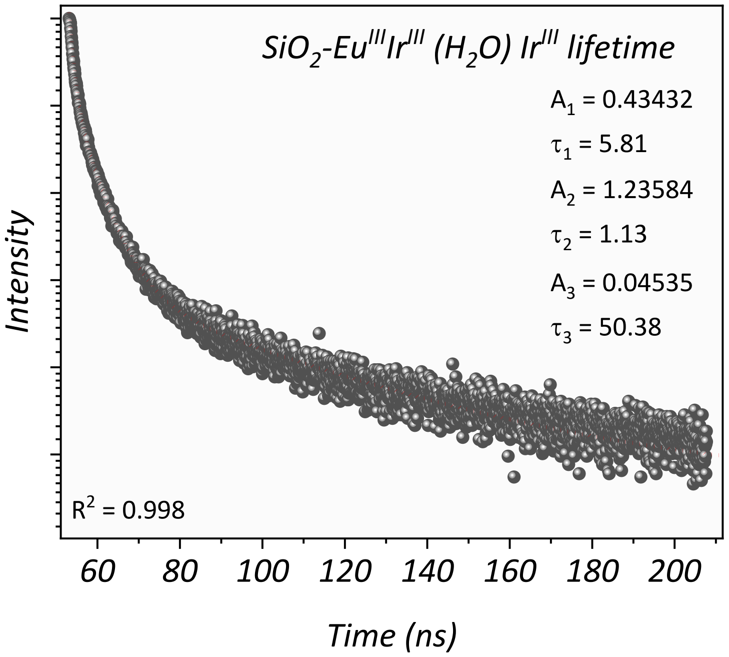

Figure S12. Emission lifetime of Ir^{III} component in SiO_2-$Eu^{III}Ir^{III}$ sample in aqueous suspension, obtained upon excitation at 375 nm and emission at 558 nm.

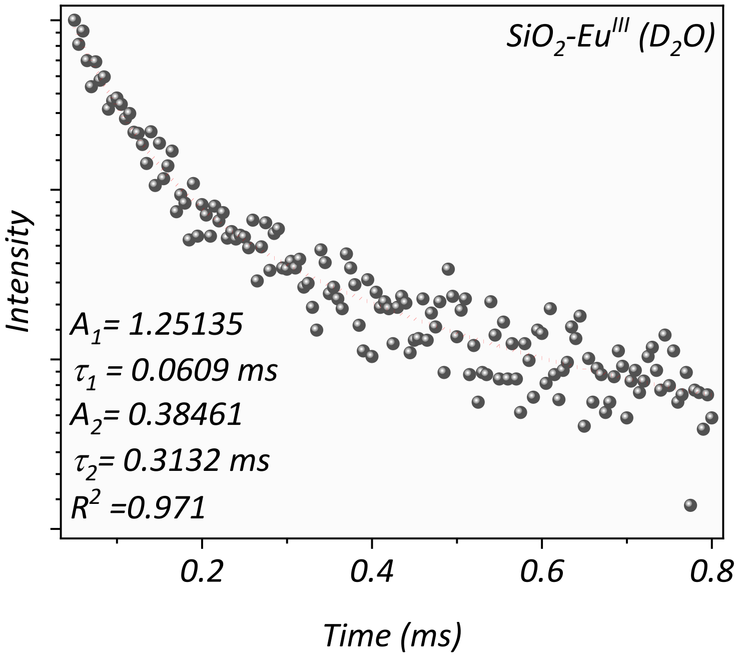

Figure S13. Emission lifetime of Eu^{III} in SiO_2-Eu^{III} sample in deuterated aqueous suspension, obtained upon excitation at 393 nm and emission at 617 nm.

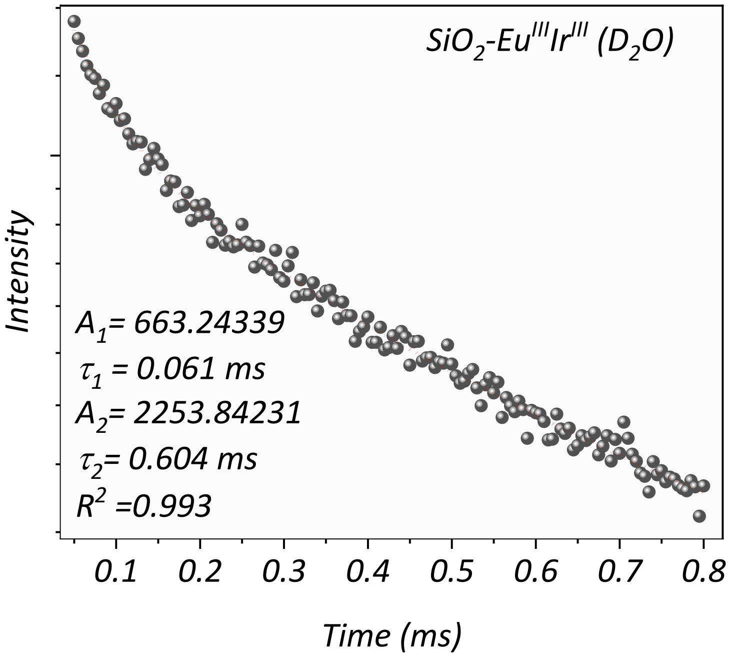

Figure S14. Emission lifetime of Eu^{III} in SiO_2-$Eu^{III}Ir^{III}$ sample in deuterated aqueous suspension, obtained upon excitation at 393 nm and emission at 617 nm.

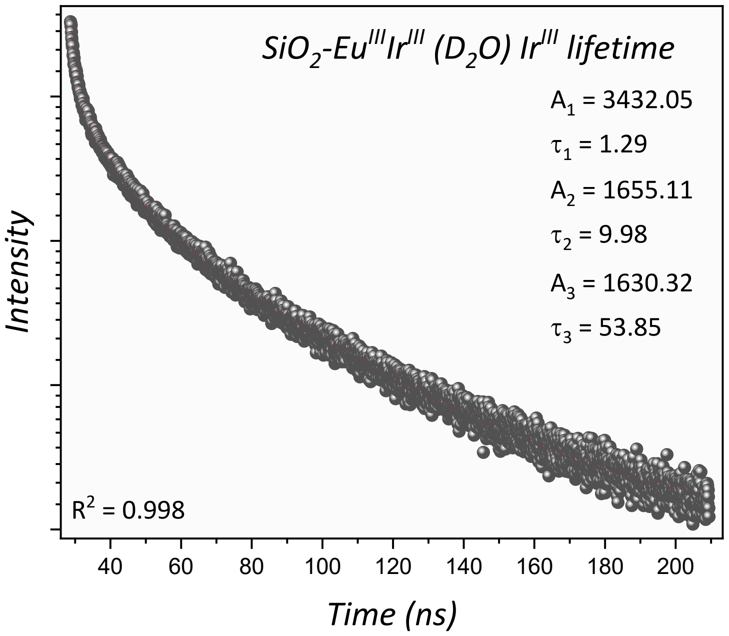

Figure S15. Emission lifetime of Ir^{III} component in SiO_2-$Eu^{III}Ir^{III}$ sample in deuterated aqueous suspension, obtained upon excitation at 375 nm and emission at 558 nm.

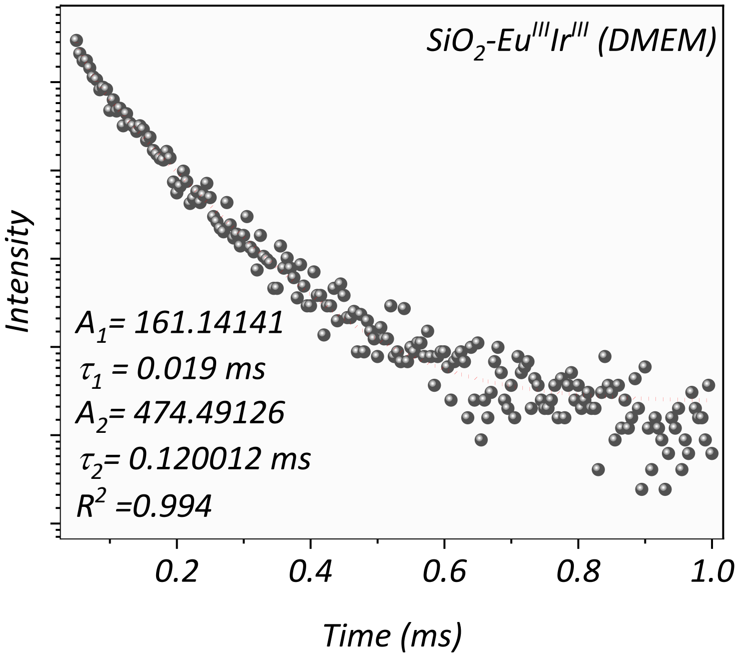

Figure S16. Emission lifetime of Eu^{III} in SiO_2-Eu^{III} sample in DMEM suspension, obtained upon excitation at 393 nm and emission at 617 nm.

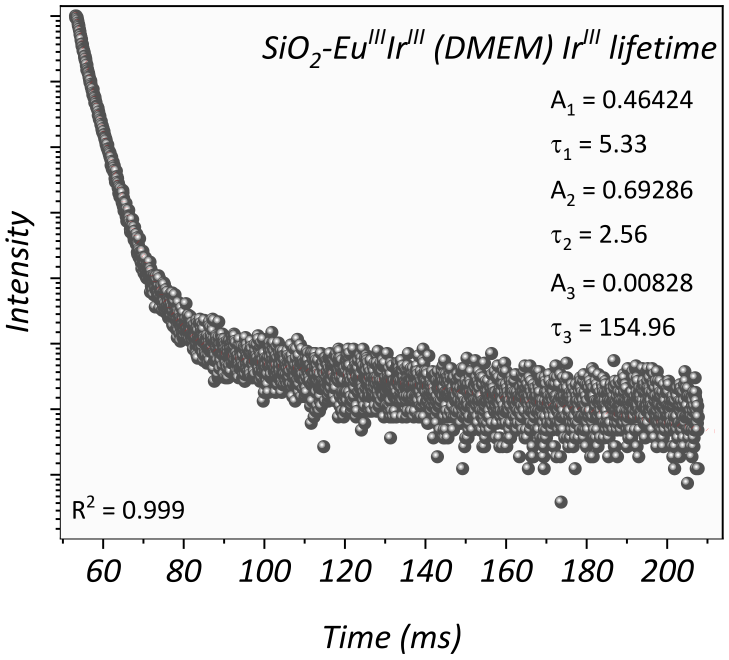

Figure S17. Emission lifetime of Ir^{III} component in SiO_2-Eu^{III}Ir^{III} sample in DMEM suspension, obtained upon excitation at 375 nm and emission at 558 nm.

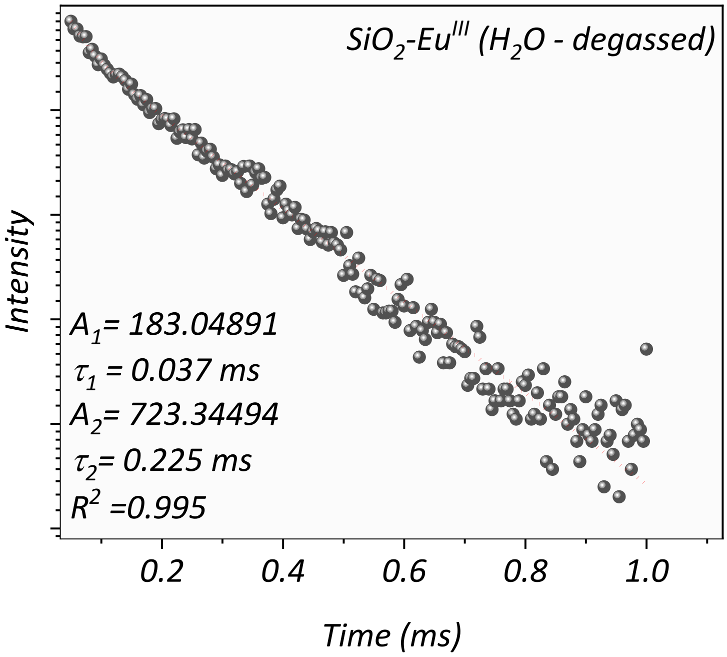

Figure S18. Emission lifetime of Eu^{III} in SiO_2-Eu^{III} sample in degassed aqueous suspension, obtained upon excitation at 393 nm and emission at 617 nm.

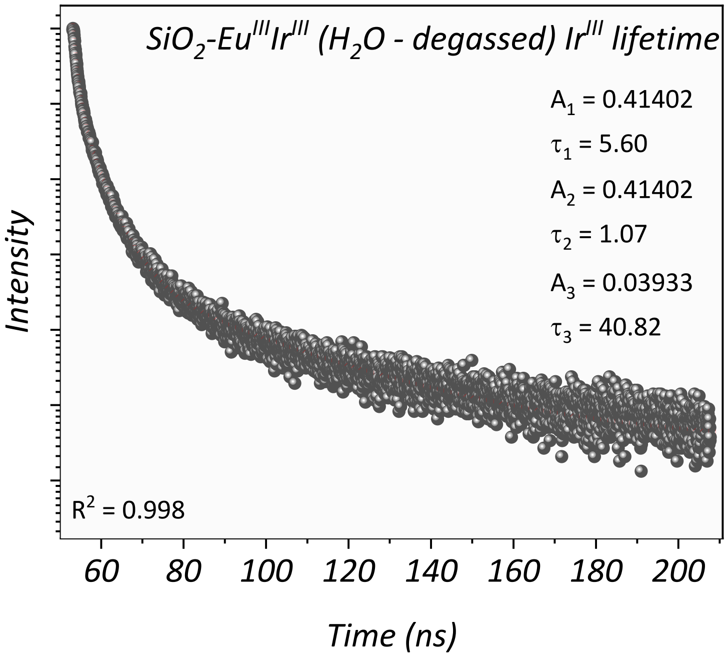

Figure S19. Emission lifetime of Ir^{III} component in SiO_2-Eu^{III}Ir^{III} sample in degassed aqueous suspension, obtained upon excitation at 375 nm and emission at 558 nm.

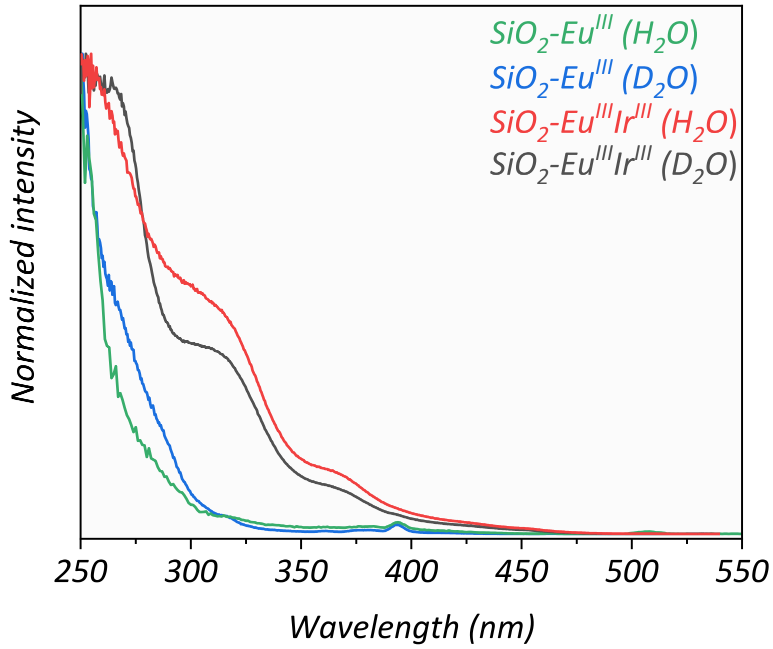

Figure S20. Excitation spectra of SiO_2-Eu^{III} and SiO_2-Eu^{III}Ir^{III} samples measured in aqueous and deuterated aqueous suspension. (λ_{em} 617 nm)

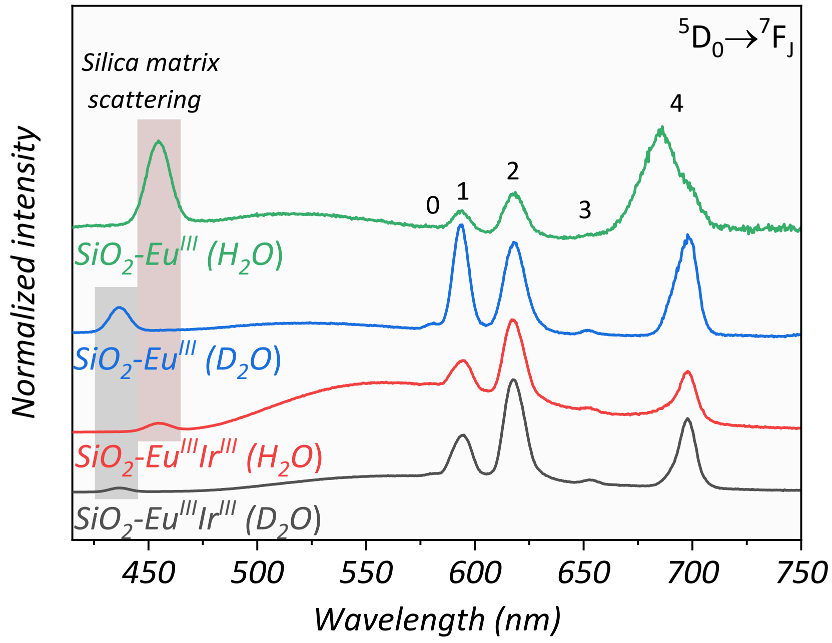

Figure S21. Emission spectra of SiO_2-Eu^{III} and SiO_2-Eu^{III}Ir^{III} samples measured in aqueous and deuterated aqueous suspension. (λ_{ex} 393 nm)

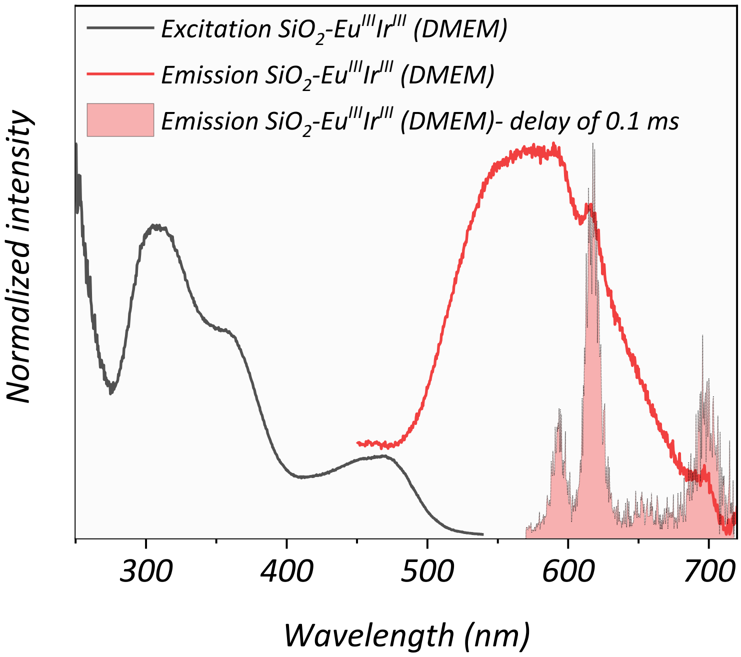

Figure S22. Excitation and Emission spectra of SiO_2-$Eu^{III}Ir^{III}$ samples measured in DMEM suspension. (λ_{em} 617 nm and λ_{ex} 393 nm)

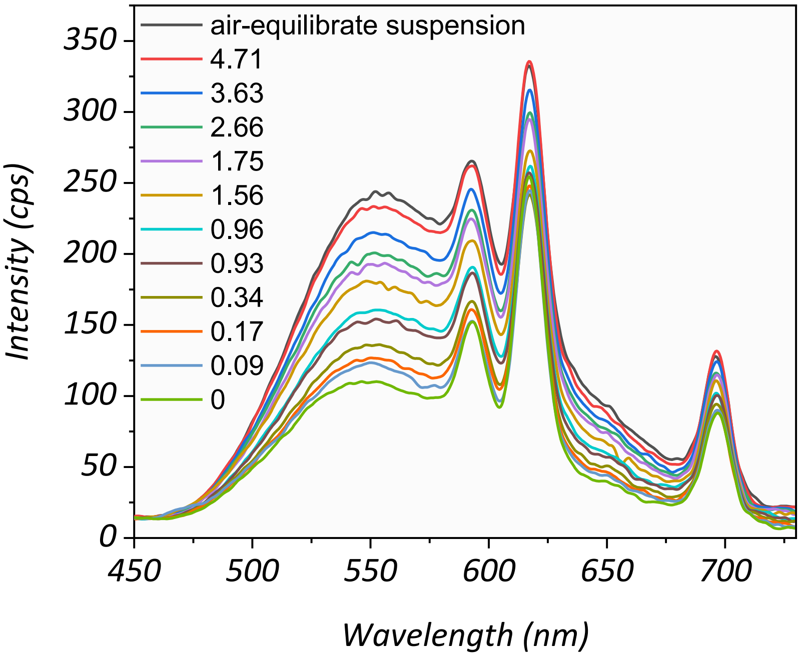

Figure S23. Raw emission spectra obtained at different dissolved oxygen (DO) concentrations.

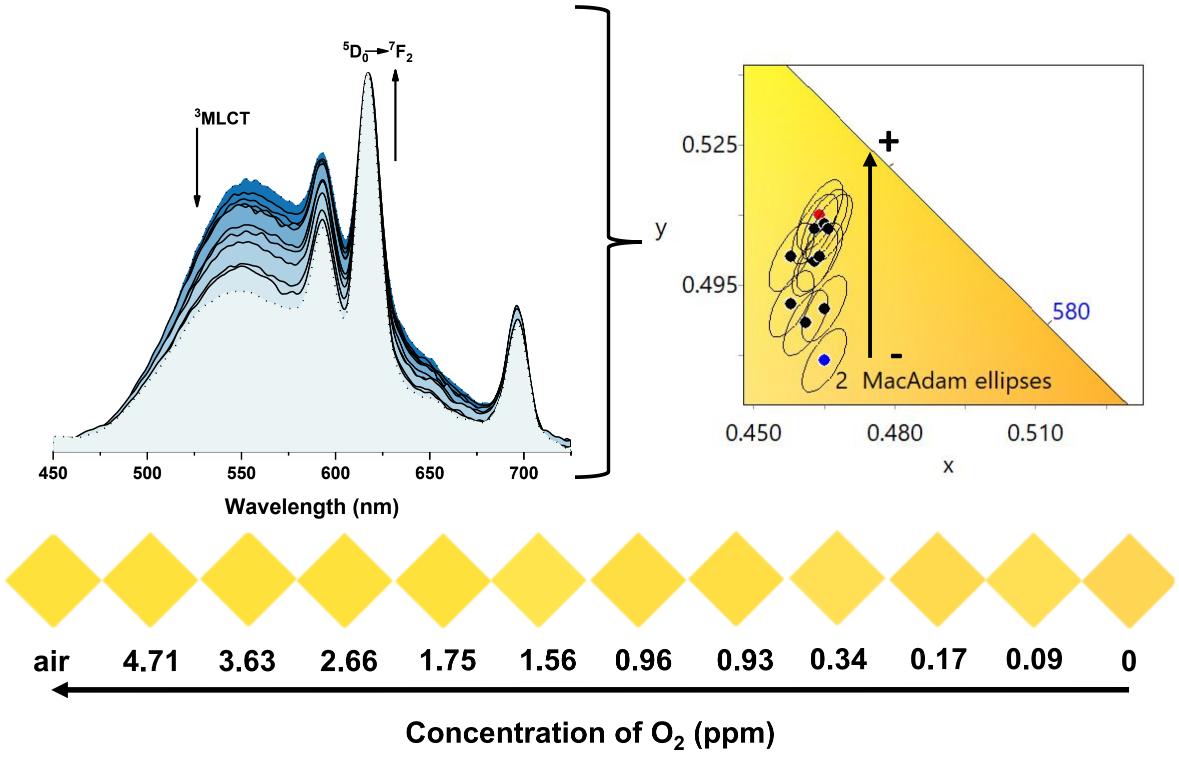

Figure S24. Variation in the color perception represented by CIE 1931 2° color coordinates.

SUPPLEMENTARY INFORMATION – SECTION II

The experimental lifetime (τ) of an excited state is determined by monitoring the luminescence decay as a function of time, which corresponds to the duration required for the emissive state to decay to 1/e of its initial intensity. The depopulation process is described by Equation S1, where the emission intensity is directly proportional to the population of electrons in the excited state.

$$I = I_0 \cdot e^{-t/\tau} \text{ (Equation S1)}$$

Depopulation can occur by radiative (A_r) or nonradiative (A_{nr}) pathways. The total decay rate ($A_t = A_r + A_{nr}$) is expressed as the reciprocal of the lifetime, as demonstrated in Equation S2[1]:

$$\tau^{-1} = A_r + A_{nr} \text{ (Equation S2)}$$

The radiative decay rate is determined by summing the photons emitted in each of the $^5D_0 \rightarrow {}^7F_J$ (J = 0, 1, 2, 3, and 4) transition sets, as outlined in Equations S3 and S4:

$$A_r = A_{00} + A_{01} + A_{02} + A_{03} + A_{04} \text{ (Equation S3)}$$

$$A_{0J} = A_{01} \left(\frac{S_{0J}}{S_{01}}\right)\left(\frac{\nu_{01}}{\nu_{0J}}\right) \text{ (Equation S4)}$$

A_{01} represents the spontaneous decay rate associated with the $^5D_0 \rightarrow {}^7F_1$ transition, which is employed as an internal standard since it is allowed by the magnetic dipole transition and is unaffected by the surrounding electric field. $S_{01:0J}$ and $\nu_{01:0J}$ denote the area and barycenter of the $^5D_0 \rightarrow {}^7F_1$ and $^5D_0 \rightarrow {}^7F_J$ transitions (J = 0, 2, 3, and 4), respectively. By utilizing both the radiative and nonradiative decay rates, the intrinsic emission quantum yield (Φ_{Eu}^{Eu}) of the $^5D_0$ emissive state of the complex can be determined, as expressed in Equation S5[1]:

$$\Phi_{Eu}^{Eu} = \frac{A_r}{A_r + A_{nr}} \text{ (Equation S5)}$$

SUPPLEMENTARY INFORMATION – SECTION III

The estimation of the functionalization steps and the C/N atomic ratio on the particles was performed based on elemental analysis measurements, as described below.

- For SiO_2-COOH sample.

The experimental carbon content was determined to be 3.13%. Considering a 100 g sample, this corresponds to 3.13 g of carbon. The number of moles of carbon (n_C) in the sample can therefore be calculated using the following relationship:

$$n_C = \frac{m_C}{MM_C} = \frac{3.13g}{12.01\ g\ mol^{-1}} = 0.260\ mol$$

where $m_C$represents the mass of carbon in the sample and MM_Ccorresponds to the molar mass of carbon.

The same procedure was applied for nitrogen. The experimental nitrogen content was determined to be 0.72%. Thus:

$$n_N = \frac{m_N}{MM_N} = \frac{0.72g}{14.007\ g\ mol^{-1}} = 0.0514\ mol$$

Knowing the number of moles of carbon and nitrogen, the experimental carbon-to-nitrogen ratio $(R_{C/N})$can be determined as follows:

$$R_{C/N} = \frac{n_C}{n_N} = \frac{0.260\ mol}{0.0514\ mol} = 5.09$$

The theoretical carbon-to-nitrogen ratio $(R_{C/N})$can be determined by considering a single functionalization chain, as follows:

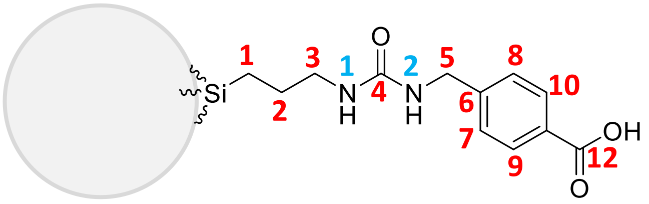

$$R_{C/N} = \frac{n_C}{n_N} = \frac{12}{2} = 6$$

This result indicates that the experimental value (5.09) is close to the initially proposed one (6.0).

The same procedure was applied to the SiO_2-Eu^{III} and SiO_2-Eu^{III}-Ir^{III} samples, and the calculated values are summarized in Table S2.

Table S2. Percentage values and experimental (exp.) and calculated (calcd.) C/N ratio ($R_{C/N}$) for SiO_2-Eu^{III}, and SiO_2-$Eu^{III}Ir^{III}$. n is the mol number of each element found experimentally.

Sample		exp. %	n	exp. $R_{C/N}$	calcd . $R_{C/N}$
SiO_2-Eu^{III}	C	1.75	0.1457	8.18	SiO_2-Eu^{III} (without ethanol); 6.0
	N	0.25	0.0178		SiO_2-$Eu^{III}(C_2H_5OH)_2$; 8.0
SiO_2-$Eu^{III}Ir^{III}$	C	2.84	0.2364	6.03	SiO_2-$Eu^{III}Ir^{III}$ (2 Ir^{III} Complex); 8
	N	0.55	0.0392		SiO_2-$Eu^{III}Ir^{III}$ (1 Ir^{III} Complex); 7.7

The degree of functionalization was determined for the SiO_2-COOH sample based on the experimental carbon and nitrogen contents. Considering that each functionalization chain contains 12 carbon atoms ($N_{C\text{ in one COOH chain}}$), the number of carboxylic chains (n_{COOH}) in 100 g of sample can be calculated as follows:

$$n_{COOH} = \frac{n_C}{N_{C\text{ in one COOH chain}}} = \frac{0.260\text{ mol}}{12\text{ C}} = 0.0216\text{ mol}$$

To determine the number of carboxylic chains per gram of sample, the following assumption was considered:

0.0216 mol of COOH chain in 100 g of the sample, thus in 1 g the amount is equal $2.1\text{x}10^{-4}$ mol g^{-1} (0.21 mmol g^{-1}). The same was made by using the percentage of nitrogen atom, and the functionalization degree was found to be 0.25 mmol g^{-1}.

[1] Binnemans, K. (2015). Interpretation of europium (III) spectra. *Coordination Chemistry Reviews*, *295*, 1-45. (https://doi.org/10.1016/j.ccr.2015.02.015)
